# Prediction of Protein–Protein Binding Interactions in Dimeric Coiled Coils by Information Contained in Folding Energy Landscapes

**DOI:** 10.3390/ijms22031368

**Published:** 2021-01-29

**Authors:** Panagiota S. Georgoulia, Sinisa Bjelic

**Affiliations:** 1 Department of Chemistry and Molecular Biology, University of Gothenburg, 405 30 Gothenburg, Sweden; panagiota.georgoulia@gu.se; 2 Department of Chemistry and Biomedical Sciences, Linnaeus University, 391 82 Kalmar, Sweden

**Keywords:** coiled coils, folding energy landscapes, ROSETTA, SYNZIPs, protein–protein interactions

## Abstract

Coiled coils represent the simplest form of a complex formed between two interacting protein partners. Their extensive study has led to the development of various methods aimed towards the investigation and design of complex forming interactions. Despite the progress that has been made to predict the binding affinities for protein complexes, and specifically those tailored towards coiled coils, many challenges still remain. In this work, we explore whether the information contained in dimeric coiled coil folding energy landscapes can be used to predict binding interactions. Using the published SYNZIP dataset, we start from the amino acid sequence, to simultaneously fold and dock approximately 1000 coiled coil dimers. Assessment of the folding energy landscapes showed that a model based on the calculated number of clusters for the lowest energy structures displayed a signal that correlates with the experimentally determined protein interactions. Although the revealed correlation is weak, we show that such correlation exists; however, more work remains to establish whether further improvements can be made to the presented model.

## 1. Introduction

Coiled coils are the simplest form of a protein–protein interface with two or more alpha helices supercoiling around each other to form homo- or heterocomplexes of parallel or antiparallel helical bundles [1]. The stable complexes of the dimeric coiled coils have been predicted to exist from as long ago as the 1950s by Francis Crick [2], and are found in ~10% of eukaryotic proteins [3]. The hallmark of regular left-hand twisted coiled coils is the heptad repeat, wherein the first and fourth position in the pattern is occupied by hydrophobic core residues tightly packing into what is widely known as knobs-into-holes interactions [4,5,6,7]. The rules governing this ubiquitous coiled coil structural motif have been extensively studied; for example, the identity per se of the interface residues occupying positions *a* and *d* seems to determine the oligomeric state, whereas imposition of polar residues at opposing *a*-positions lead to a parallel coil assembly. The simplicity and extreme stability of the coiled coils, though highly versatile, have rendered them ideal oligomerization tags. They are implicated in a variety of diverse functions, mainly as driving assembly in motor proteins, ion channels, and transcription factors, but also leading to structure formation in fibrous proteins [1]. In addition, they are found in viral glycoproteins facilitating the entry via protein–protein interactions with host cell receptors. Coiled coils have been extensively studied in protein folding and were one of the first successful examples of structure prediction algorithms [8]. Besides the sequence-to-structure heptad-based approaches in designing coiled coils, additional methodology was developed ranging from semi-empirical approaches to computational design utilizing Crick parametric equations [9,10,11,12]. Coiled coils have been the workhorse in protein engineering all the way to novel protein designs with desired functions; understanding the rules governing the coiled coil folding and assembly is thus central to many important research areas.

Rosetta protein modelling software has been successfully used both to fold coiled coil complexes and to design new protein topologies de novo. The methodology allows for the modelling of non-ideal features, register shifts, and alternate states which are not straightforwardly accessible to rational parametric approaches. For example, the Rosetta implemented “fold’n’dock” method [13,14] allows for simultaneous folding and docking of individual alpha helical subunits followed by all-atom refinement using the Rosetta energy function [15]. The Rosetta energy terms are based on both physics-based potentials as well as terms derived from statistical analysis of experimentally solved structures deposited in the PDB (cf. Leaver-Fay et al. and Alford et al. [16,17] for detailed description of the Rosetta energy terms). Starting from the sequence input of the monomeric chains, fold’n’dock uses the Monte Carlo algorithm to sample alternative conformations guided by the energy terms. An important aspect of the coiled coil prediction here is achieved from local structural preference stored in the 3-mer and 9-mer backbone fragments randomly inserted into the structure to generate a single conformation. The modelled, individual subunits are then docked together to form a complex; the process also includes the simultaneous rotameric sampling of amino acid side chains. The cycle is repeated until a sufficiently large number of models are generated to describe the folding energy landscape accessible by the sampling algorithm and evaluated by the energy function.

The described Rosetta fold’n’dock methodology has been reliable in modelling the oligomerization preference in coiled coils with a prediction success rate of coiled coil topologies being quite high for both homo- and heterodimeric complexes. The accuracy of predicting the correct topology of homodimers from the first principles was about 90% [14], with data indicating almost as good a recovery for heterodimers with the accuracy of 82% [18]. The challenge in accurately predicting these coiled coil structures compared to other protein–protein complex formations lies in discriminating among the isoenergetic models separated by low energy barriers from the conformationally distinct native structure; for example, the parallel and antiparallel topologies of coiled helical subunits. In homodimeric coils, there is the additional complexity of predicting the correct oligomerization state through interactions of two or more subunits, forming complexes. When it comes to heterodimeric coiled coils, there are further criteria of discrimination between homo and hetero oligomerization preferences in dimer formation, which has been shown to be of the same energy magnitude for certain cases [18]. Finally, coiled coils that are part of naturally occurring complexes may have tendencies to form flat energy landscapes and may require folding energy from a whole three-dimensional complex to properly assemble. Moreover, the similarity in the packing interactions is interpreted in isoenergetic intermediates in these flat folding landscapes.

Herein, we utilize the current methodology to investigate to what extent the shape of folding energy landscape from in silico simulations can be coupled to the prediction of experimental protein–protein binding affinities. This is the most challenging of the prediction tasks, because modelling starts from amino acid sequences that form protein–protein complexes without assuming any quaternary structure of the complex. In contrast, the often-built predictive models for binding are based on pre-existing, experimentally solved protein complexes. Nevertheless, the information derived from the folding landscapes contains information that is relevant for folding, for example the existence of competing topological preferences or alternative near-native energetic states. The energetics of interface formation are thus inferred by the information additionally contained in the energy landscapes, and therefore we can explore how this information may be used to assess the binding energies that guide the complex formation.

The method is applied on a dataset reported by Reinke et al., consisting of 48 coiled coil monomers [19] called SYNZIPs that are on average 46 amino acids long with a standard deviation of six amino acids. These were originally designed to interact with leucine-zipper regions of human transcription factor but have been further assessed for pairwise interactions amongst themselves. The binding interactions [19] were determined using a microarray assay and correlated here to the binding energies from mutually folded SYNZIPs pairs, as both homo- and heterodimers. The SYNZIPs formed complexes consisting of 90 ± 11 amino acids (mean ± SD). The comparison of resulting terms derived from 1176 folding energy landscapes with the experimental binding dataset showed a medium correlation between the binding signal and the number of structural clusters formed by the lowest folded energy models for each energy landscape. These results indicate that metrics based on the counting of states may increase the accuracy of the design predictions, in comparison to those relying only on energetics.

## 2. Results

The experimental microarray values reported in Table S2 in the Supplementary Materials by Reinke et al. [19] were used as experimental binding values for SYNZIP coiled coils. They were reported as fluorescence signals, measured twice for each dimeric interaction (depending on the array immobilized coil); therefore, we used the array fluorescence binding response normalized to the maximum signal for the average signal. Additionally, signals greater than 50 fluorescence units (and up to 49,000 representing the strongest interactions) were used for data fitting which resulted in a total of 985 data points out of 1176 used for initial model building (cf. Section 4).

### 2.1. Modelling Folding Energy Landscapes of Dimeric Coiled Coils

Rosetta fold’n’dock application was used to fold monomeric coiled coils subunits into homo- and heterodimers. Each model was calculated by starting from the amino acid sequences of the monomers which were folded by fragment insertion and simultaneously docked to give a dimeric coiled coil complex. The calculations were repeated 20,000 times for each of the dimers to appropriately sample their folding energy landscape. The dataset consisted of 48 SYNZIP monomers, therefore it was necessary to calculate 1176 folding funnels, with a cumulative sum of 24 million models for all dimers (cf. Supplementary Materials: data-folding energy landscapes of SYNZIP coiled coils). For each of the dimers, the lowest energy model was selected as the solution of the folded complex and all other models were plotted as the function of the total energy versus the root mean square deviation (RMSD) for the C_α_ atoms from that model. The resulting plots represent the typical folding energy landscapes for proteins, wherein the lowest energy model often occupies a well-defined folding well (cf. Supplementary Materials). Additionally, the landscapes showed the characteristics of dimeric coiled coils, where parallel and antiparallel topologies often have similar energetics, giving rise to an additional low energy folding well at high RMSD from the lowest energy model.

This demonstrated the validity of the used methodology in the simulation of coiled coils starting from their primary structures. In all cases, two funnels were present (at low and high RMSD values, respectively), accordingly to the previous observations that both parallel and antiparallel topologies lead to defined low energy minima.

### 2.2. Energetics of the Highest Affinity Coiled Coil Dimers and Comparison to Experimental Structures

**Figure 1:**
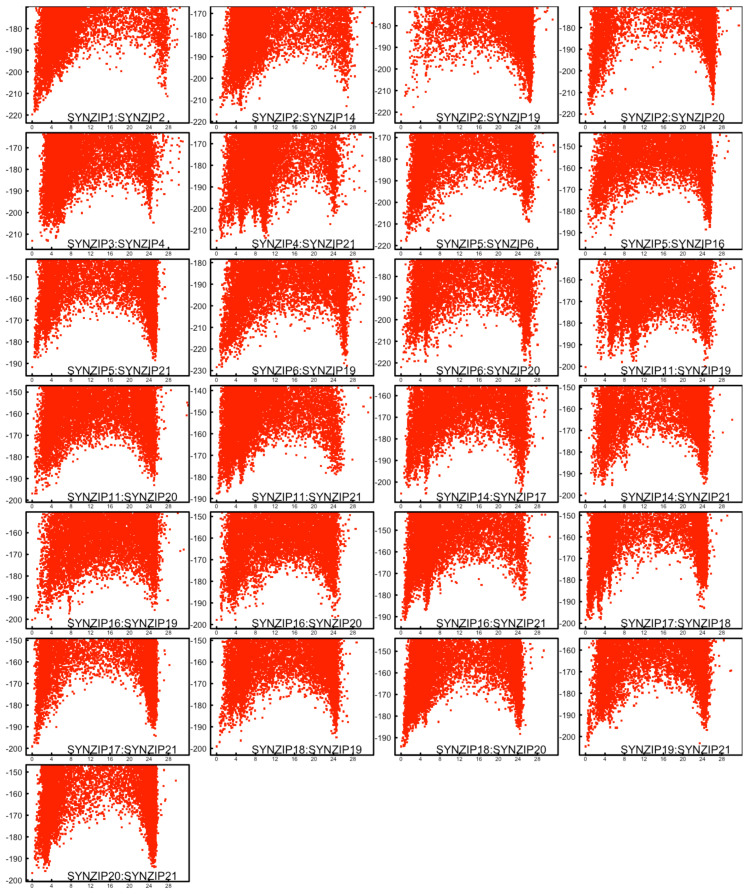
Folding funnels for highest affinity SYNZIP dimers. In each plot, the energy for the final folded structure from Rosetta fold’n’dock sampling was plotted as a function of root mean square deviation (RMSD) from the lowest energy model (calculated in Ångström over corresponding C_α_ atoms). In total, 20,000 folding trajectories were simulated, thus the plots show energy vs. RMSD for 20,000 structures. Out of the 25 energy landscapes for the highest affinity dimers, almost all pairs show well defined folding funnels, except for SYNZIP4:SYNZIP21 and SYNZIP11:SYNZIP19 complexes.

Before further investigation of any correlation between the folded dimeric coiled coils and the published binding dataset, we checked the performance of the fold’n’dock methodology on the SYNZIP pairs for which extensive experimental data existed. For example, the SYNZIP pairs in Table 1 have been experimentally characterized with respect to binding dissociation constants besides the published array binding data [20]. In addition, for the two of the coils, SYNZIP1:SYNZIP2 and SYNZIP5:SYNZIP6, there were experimentally solved crystal structures available [19]. The SYNZIP pairs in Table 1 have binding dissociation constants lower than 30 nM, therefore they would be expected to have well-defined energy landscapes with sharp folding funnel minima towards the predicted lowest energy structure. Out of these 25 modelled coiled coils, SYNZIP4:SYNZIP21 and SYNZIP11: SYNZIP19 display alternate folding states up to 12 Å RMSD from the lowest energy model (Figure 1), similarly to what has been observed previously for coiled coils and protein complexes [14,15,18]. The folding landscapes for other pairs in Table 1 are quite well-defined, as shown in Figure 1. In the case of SYNZIP2: SYNZIP19, the sampling around the lowest energy model seems to be sparser in comparison to the opposite topology which indicated challenges with sampling.

**Figure 2:**
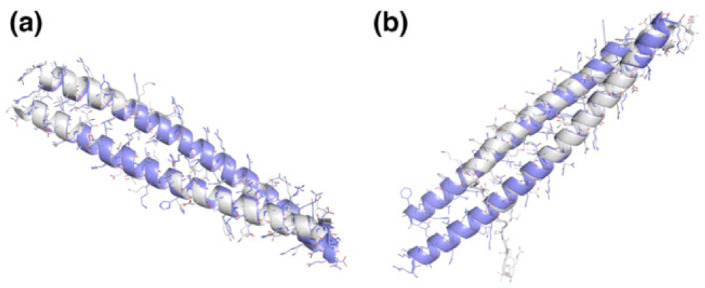
In two cases of modelled coiled coils (purple) there are experimentally solved crystal structures (gray). (**a**) SYNZIP1:SYNZIP2 the lowest energy model fully recovered the crystal structure. (**b**) SYNZIP5:SYNZIP6 frameshifted in one of the coiled coils at the formed interface. Nevertheless, both folded energy landscapes indicate a well-formed folding funnel towards the lowest energy model.

For the SYNZIP1:SYNZIP2 pair, for which there is an experimentally solved structure (PDB ID 3he5), the theoretical model perfectly captures the native interactions with C_α_ RMSD of 0.63 Å (Figure 2a). In the case of SYNZIP5:SYNZIP6, the experimental structure of the complex (PDB ID 3he4) and the model do not agree because there seems to be a part of the coil that is disordered due to the crystal contacts (Figure 2b). These crystal contacts may unravel the coil, which leads to formation of interactions not found in the theoretical model, where it prefers to form interactions with its partner coil model (because only a dimeric oligomerization state was simulated).

**Figure 3:**
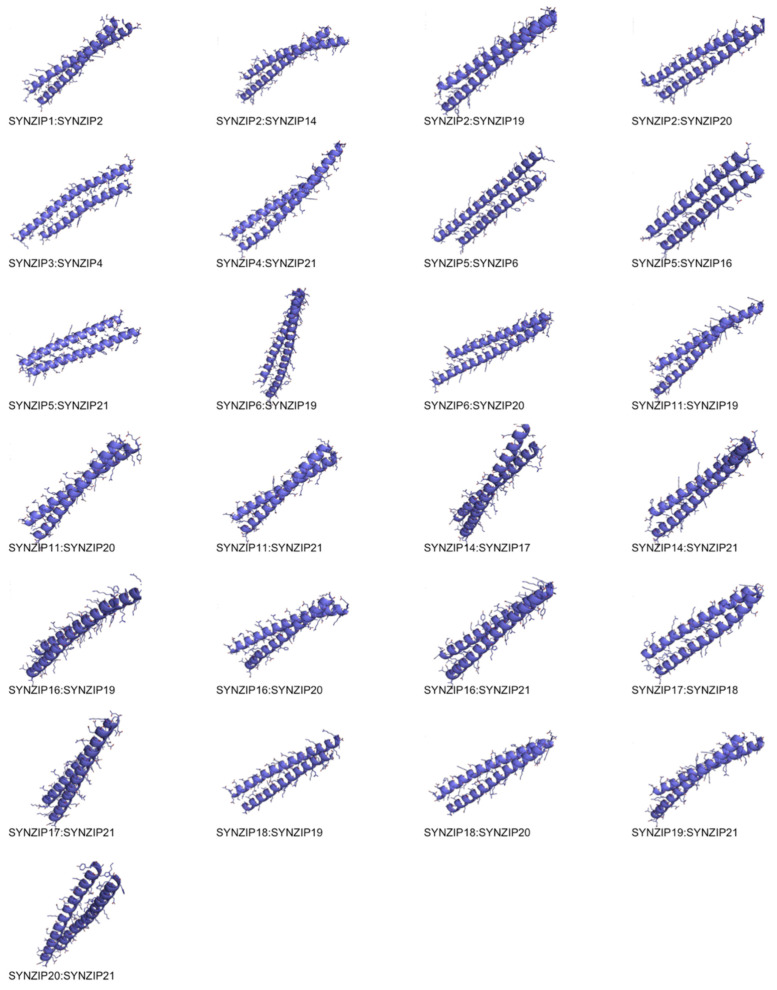
Lowest energy models for the highest affinity coiled coils indicating well-formed complexes.

Structurally, the lowest energy models show the characteristic interactions of the coiled coil complexes where each subunit is interacting along the longitudinal axis (Figure 3). The coiled coil proteins in Figure 3 and Table 1 have also been characterized experimentally with respect to their topology, which we recovered at 68%.

### 2.3. Characteristics of the Energy Landscapes and Properties of the Lowest Energy Models

**Figure 4:**
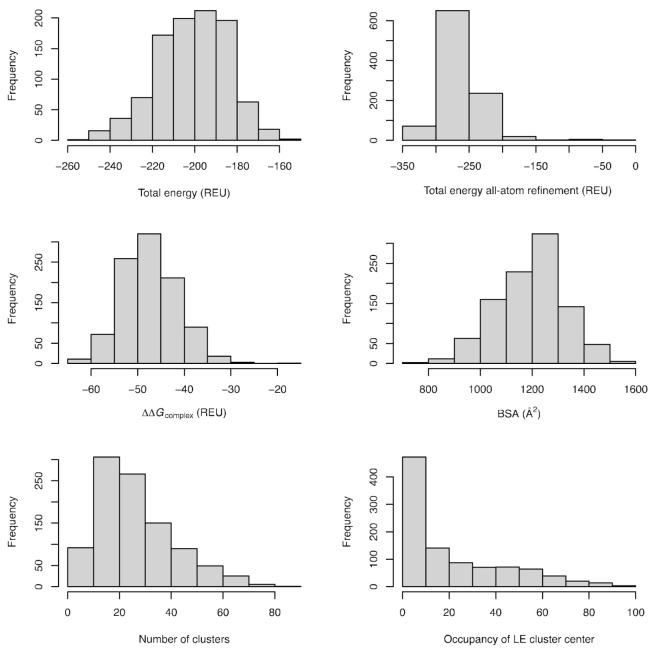
Preliminary assessment of the folded complexes for each coiled coil pair. The plots show several calculated metrics: the lowest total energy, the lowest total energy after all-atom refinement, the energy of complex formation, the buried surface area (BSA) upon interface formation, the number of clusters for the corresponding one hundred lowest energy models, and occupancy of the lowest energy (LE) cluster center.

Physics- and statistics-based terms that have been shown in general to have correlation with binding energies were investigated for 985 coiled coils with an arrayscore greater than 50. We folded and docked the individual coils; therefore, their total energy should be a representative measure of the binding interaction. The histograms of the total energy of the system for all coiled coils pairs from the fold’n’dock simulations are presented in Figure 4. The total energy is dependent on the number of amino acids and therefore the number of possible interactions formed, but overall, it accurately represents the dataset with mean ± SD values of −201 ± 23 Rosetta energy units (REU). Additionally, we calculated the energetics after all-atom minimization of the 100 lowest energy structures to investigate whether additional sampling that often leads to lower energy structure may influence binding prediction. After the refinement, we observed a significant drop in total energy for most of the structures, which was now on average 60 REU lower, at −260 ± 32 REU.

In our calculation, we included the most traditionally used predictors of binding; for example, the total energy of the complex after subtracting the energies of the individual subunits. The values for this term, ∆∆*G*_complex_, were in the range of −47 ± 6 REU, indicating that the complex formation according to the energy function used was favorable. Buried surface area (BSA) upon complex formation was also determined, because it has been shown to also correlate with binding free energies. The calculated values of 1200 ± 130 Å^2^ indicate formed complexes that are in line with the BSA values determined for naturally occurring protein complexes [21].

We also wanted to use the information contained in the energy landscapes, thus clustering of the 100 lowest energy structures was carried out for each of the dimers. This term determines how far these low energy models are structurally from each other, where well-defined energy landscapes with sharp folding funnels should have fewer clusters than landscapes with multiple minima that behave iso-energetically. On average, there are *N*_clust_ = 26 ± 15 at 2 Å clustering cut-offs, with the largest distribution of coiled coils having clusters between 10–30 at the used threshold. It is worth noting that the coiled coils with well-defined folding funnels may still exhibit a large absolute number of clusters due to the fact that the energy vs. RMSD were calculated only towards the lowest energy model, while clustering was performed mutually between 100 models. In a sense, the dimensionality of the plotted landscapes was reduced. We also sorted the clusters according to the energy of the cluster center, to investigate whether occupancy of the lowest energy cluster center may contain any additional information relevant for binding prediction. The lowest energy cluster centers had the fewest number of models, i.e., the lowest occupancy, according to the histogram presented in Figure 4.

### 2.4. Prediction of the Coiled Coil Binding Interactions

To investigate the correlation between the coiled coil binding interactions published as array binding data [19] and the different variables discussed above, a multiple linear regression (as implemented in the statistics software package R [22]) was carried out. Only coiled coils with a positive array binding signal greater than 50 were included in the statistical fitting; the binding data were treated similarly as in the original publication, in which the measured array fluorescence binding response was normalized to the maximum signal according to the following equation:

(1) $$arrayscore=\;-\ln\frac{array\;fluorescence\;binding}{{\left(array\;fluorescence\;binding\right)}_{max}}$$

**Figure 5:**
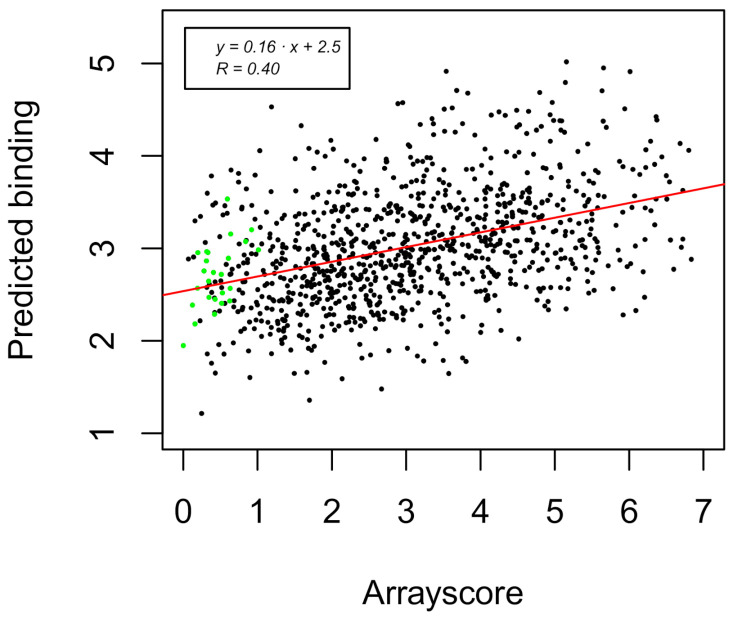
Predicted values for dimeric coiled coil formation binding as a correlation to the calculated arrayscore from fluorescence binding responses (low values indicate binding, while high values represent non-interacting coiled coil dimers). The line in the plot corresponds to the fit of calculated vs. experimental values; the green points mark the prediction of 25 coiled coils with determined *K*_d_ values.

Next, the arrayscore was correlated to total energy of the system, total energy after all-atom refinement, ∆∆*G*_complex_, BSA, number of clusters, and occupancy of the lowest energy cluster center. The best correlation was obtained for the total energy of the system, ∆∆*G*_complex_, and number of clusters according to the plot of predicted binding vs. experimental arrayscore, as shown in Figure 5. The included terms were found to be highly significant, while the other tested terms presented in the histograms in Figure 4 did not show any correlation to the experimental data.

**Figure 6:**
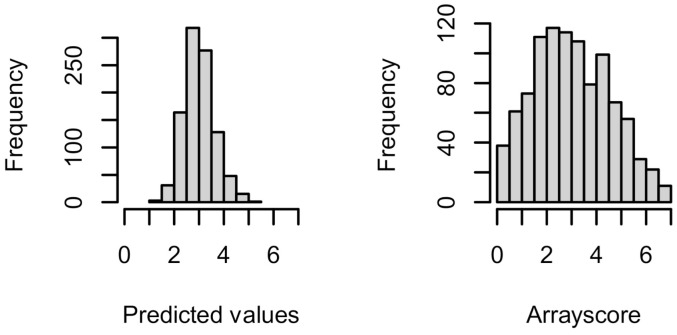
Distribution of the predicted and the experimental binding (arrayscore) values.

Decomposition of the individual fitted terms show that the number of clusters is the most significant variable with Pearson’s correlation coefficient (*R*_Pearson_) equal to 0.32, with the calculated binding ∆∆*G*_complex_ being somewhat lower, whereas the total energy is only weakly correlated with *R*_Pearson_ = 0.19. Combining the individual variables leads to a higher correlation, but only when the number of clusters was fitted together with the either the total energy terms or the ∆∆*G*_complex_. Finally, when all three terms were tested together (Table 2), we obtained the highest correlation of *R*_Pearson_ = 0.4 with a *p*-value < 0.001 for the tested variables. Although the model statistically captured the correlation between the experimental and predicted values, there is some information lacking, as demonstrated by the distribution of predicted and experimental values in Figure 6. Both distributions are centered at a mean value of 3.0 but differ in standard deviations 0.61 and 1.5 for predicted and experimental values, respectively. The model thus did not capture the low-affinity binders and those that did not interact.

## 3. Discussion

While the overall structure of dimeric coiled coils can be described by simple parametric equations, the absolute binding free energy between the two monomers cannot be theoretically calculated with high reliability by existing methods. The binding affinity between two proteins is determined by Gibbs free energy, which is related to the experimental equilibrium dissociation constant according to the correlation ∆*G*_bind_ = −RT ln *K*_d_. This has led to an increasing interest towards the development of predictive methods that rely on calculations of differences between the binding free energies, i.e., ∆∆*G*_bind_. These are traditionally calculated by methods of alchemical transformations, where only a few atoms are transformed by either free energy perturbation (FEP) or thermodynamic integration (TI) methods, and approximations thereof. These are often employed in assessing small molecule binding to protein drug targets but are also used in modelling the effect of amino acid changes on protein–protein interactions. The time-consuming nature of FEP/TI consequently steered the development towards faster methodologies grounded in statistical regression methods, where terms correlated to binding are included, ranging from buried (binding) surface area and shape complementarity, to residue contacts, number (strength) of hydrogen bonds, and nonpolar interactions, etc.

The existing statistical models that attempt to reproduce experimental binding interactions (or the differences in binding), whether it is for small molecules interacting with macromolecules or proteins forming protein complexes, are traditionally based on terms derived by fitting the experimental binding data, often *K*_d_ values, to experimentally solved structures of corresponding complexes [23,24,25,26]. This provides the opportunity to control the parameters used for data fitting in detail, and to learn which principles are governing the complex formation. However, this requires the structural knowledge of interacting partners as well as the experimental knowledge of interaction magnitude. Technology has been developed to fold interacting proteins starting from their sequences and to dock these models simultaneously within a reasonable timeframe. This opens new opportunities to investigate the guiding principles for complex formation when experimental data are limited, which is more often the situation in computational protein design and engineering.

Dimeric coiled coils are the simplest form of binding complex between proteins, and are therefore the key model system to study protein–protein interaction energetics, including the sought-after reliable prediction of binding affinity starting from the amino acid sequence of the interacting proteins. The structural modelling of coiled coil interactions may further provide a better understanding of the driving forces governing the complex formation. The experimental and theoretical data analyzed in this work allowed us to pose the following question: what is more important for the prediction of binding interactions, the lowest energy or well-defined, steep folding funnel? From the multiple linear regression of array binding data for the SYNZIP coils, we found that there was a weak correlation to both the total energy of the system and the estimated binding energy ∆∆*G*_complex_. However, the best regression fit for the individual terms was obtained when correlating the binding interactions with the number of clusters which describe the folding propensity of the coiled coil dimers, according to the folding energy landscape. Specifically, when combing the energetic terms, for the total system energy and the estimated binding energy, ∆∆*G*_complex_, with the assessment of the folding energy landscape according to the cluster analysis, the highest correlation was obtained.

The other metrics that were tested, for example, the buried surface area, usually work well if there are experimental data in the form of solved bound structures and binding affinities that are often used for fitting. When the interface is unknown, which it was during our calculations, or in the case of mutations, the buried surface is not a good descriptor. A clear example is the effect of mutations on hot spot residues that often may lead to binding, which is several orders of magnitude weaker, while the change in buried surface area remains almost identical. Alternatively, folding trajectories have been shown to contain useful information for the determination of protein–protein interactions; however, it is still difficult to obtain sufficient accuracy from such calculations to be applicable on the large scale that would be required. Here, we try to expand what is methodologically possible by collapsing the folding trajectory search space and only take into account how well the lowest energy models from the folding simulations cluster to each other. High dispersion in clusters describes not well-defined lowest energy states, directly correlating to higher entropy, and thus should lead to less favorable free energy of the complex formation.

Although the analysis above presents a simple correlation between the experimental and predicted binding interactions, it would be interesting to carry out more complex evaluations of folding trajectories; for example, by constructing interaction networks between the clusters to investigate whether any relevant information for protein–protein interaction prediction can be derived [27,28,29,30,31,32]. Alternatively, deconstructing the energy function into interaction terms based on polarity, hydrogen bonds, etc. (or similarly residue contacts) may further improve the correlation. Overall, the results indicate that metric methods which rely on the counting of states may be better descriptors than those that are solely based on the energetic terms when it comes to predicting binding from de novo simulations of protein–protein interactions, which seems to also be the case in predicting folding. Although the found correlation was weak, we have demonstrated that such correlation thus exists in folding energy landscapes of the studied system, but future work remains to establish whether the correlation can be improved.

## 4. Materials and Methods

### 4.1. Coiled Coil Data Set

The designed SYNZIP coiled coil dataset [19] was used for the prediction of protein–protein interactions. In the original publication, the experimental binding interaction between 48 SYNZIP coiled coils was determined. The SYNZIPs were designed to preferentially heteroassociate with a goal to direct assembly of signaling complexes without any leaking crosstalk between the monomeric subunits. The coiled coil sequences were 35–54 amino acids long and were designed to interact with human transcription factors bZIP. Especially during the design process, sequences were chosen to disfavor homodimerization. A sequence alignment is presented in Figure S1 in ref [19]; each of the sequences for the synthetic peptides was named following the established nomenclature. Interactions between subunits SUNZIPx and SYNZIPy that formed complexes were denoted by SYNZIP*x*:SYNZIP*y* (*x* and *y*, numbered 1–48). Here, we also used the naming with an underscore for the complexes in the Supplementary Materials, e.g., SYNZIPx_SYNZIPy, to indicate their possible interaction, because it simplified all the naming during calculations and data processing. The sequences were extracted from Figure S1 in ref [19] and used for folding calculations.

### 4.2. Estimate of the Coiled Coil Dimer Interaction

The results of the array binding assay for 48 SYNZIP coiled coil dimers as reported by Reinke et al. represented the experimental values (Table S2 in Supplementary Materials in ref [19]) for model building. These were measured as fluorescence signals corrected for the background noise and determined reciprocally for each dimeric interaction. For the measure of binding magnitude, we used the array fluorescence binding response normalized to the maximum signal for the average signal, (SYNZIPx:SYNXIPy + SYNZIPy:SYNZIPx)/2, greater than 50, according to the following equation:

(2) $$arrayscore=\;-\ln\frac{array\;fluorescence\;binding}{{\left(array\;fluorescence\;binding\right)}_{max}}$$

This was in accordance to how the data were treated in the original publication. This produced an arrayscore that varied between 0–1 for the best interacting binders and values that were >1 for the weakest complexes. These values were directly correlated to the predicted values during the evaluation of different interaction models in this study.

### 4.3. Computational Folding Simulations

The fold’n’dock protocol, as implemented in Rosetta [13,14,17], was used to simultaneously fold and dock SYNZIP pairs which resulted in a total of 1176 folding energy landscapes. The protocol used followed the setup as described in André et al. [18], according to the methodology published by Rämisch et al. [14] and Das et al. [13]. In brief, simulations start with two extended protein chains that were folded by fragment assembly and simulated annealing to build a coarse-grained model. The fragment (3-mer and 9-mer) dataset was as published in André et al. [18]. For each coiled coil dimer, 20,000 random trajectories were calculated. The resulting models were analyzed by taking the lowest energy model as the folding solution and plotting the Rosetta total energy as a function of RMSD from that model.

### 4.4. Clustering, Energy Refinement, and Analysis

Models were sorted according to the total energy and the 100 lowest energy models were clustered using the clustering application implemented in Rosetta [17]. Clustering was carried out at a radius of 2 Å. Alternatively, these models were additionally used for energy refinement with the Rosetta energy refinement protocol [15]. Correlation analysis and model fitting were carried out by multiple linear regression package as implemented in statistics software R [22], which has also been used to plot the charts. All molecular graphics images were prepared with Pymol software [33].

## 5. Conclusions

The often-used metrics applied to binding affinity recapitulations may not be directly applicable to the forward predictions of protein–protein interaction magnitude, especially when the predictions start from the protein amino acid sequences. Other metrics derived from the information within folding energy landscapes may be better predictors, as demonstrated with the correlation of the number of clusters calculated from the lowest energy models in the folding energy landscape. Such metrics should be investigated to present how the design and prediction of protein–protein interfaces could be achieved in future. It is in these challenging cases that any improvements will lead to design processes with the highest impact for the overall success rate.

## Figures and Tables

**Table 1:** Binding dissociation constant (*K*_d_) of SYNZIP dimers as reported in Thompson et al. [20].

Coiled Coil Dimer	*K*_d_ (nM)	Coiled Coil Dimer	*K*_d_ (nM)
SYNZIP1:SYNZIP2	<10	SYNZIP11:SYNZIP21	<10
SYNZIP2:SYNZIP14	<10	SYNZIP14:SYNZIP17	<10
SYNZIP2:SYNZIP19	<10	SYNZIP14:SYNZIP21	<10
SYNZIP2:SYNZIP20	<10	SYNZIP16:SYNZIP19	<10
SYNZIP3:SYNZIP4	<30	SYNZIP16:SYNZIP20	<10
SYNZIP4:SYNZIP21	<10	SYNZIP16:SYNZIP21	<10
SYNZIP5:SYNZIP6	<15	SYNZIP17:SYNZIP18	<10
SYNZIP5:SYNZIP16	<10	SYNZIP17:SYNZIP21	<10
SYNZIP5:SYNZIP21	<10	SYNZIP18:SYNZIP19	<10
SYNZIP6:SYNZIP19	<10	SYNZIP18:SYNZIP20	<15
SYNZIP6:SYNZIP20	<10	SYNZIP19:SYNZIP21	<10
SYNZIP11:SYNZIP19	<10	SYNZIP20:SYNZIP21	<10
SYNZIP11:SYNZIP20	<10		

**Table 2:** Optimization of binding interaction predictor variables based on the (multiple) linear regression. For each model, Pearson’s correlation coefficient, *R*_Pearson_, was calculated to assess the significance of the predictive power that the model may show.

Variable	Variable Name	*R*_Pearson_ (Pred. vs. Exp)
*x*_1_	Total energy	0.19
*x*_2_	∆∆*G*_complex_	0.27
*x*_3_	Number of clusters	0.32
*x*_1_ + *x*_2_		0.27
*x*_1_ + *x*_3_		0.39
*x*_2_ + *x*_3_		0.38
*x*_1_ + *x*_2_ + *x*_3_		0.40

## Data Availability

Data is contained within the supplementary material.

## Supplementary Materials

The supplementary materials can be found at https://www.mdpi.com/1422-0067/22/3/1368/s1.

## Abbreviations

BSA	Buried surface area
REU	Rosetta energy units
