# Supplementary figures and images for: Prediction of Protein–Protein Binding Interactions in Dimeric Coiled Coils by Information Contained in Folding Energy Landscapes
Source: Int J Mol Sci. 2021 Jan 29;22(3):1368. doi: 10.3390/ijms22031368 (PMC7866404; doi:10.3390/ijms22031368)

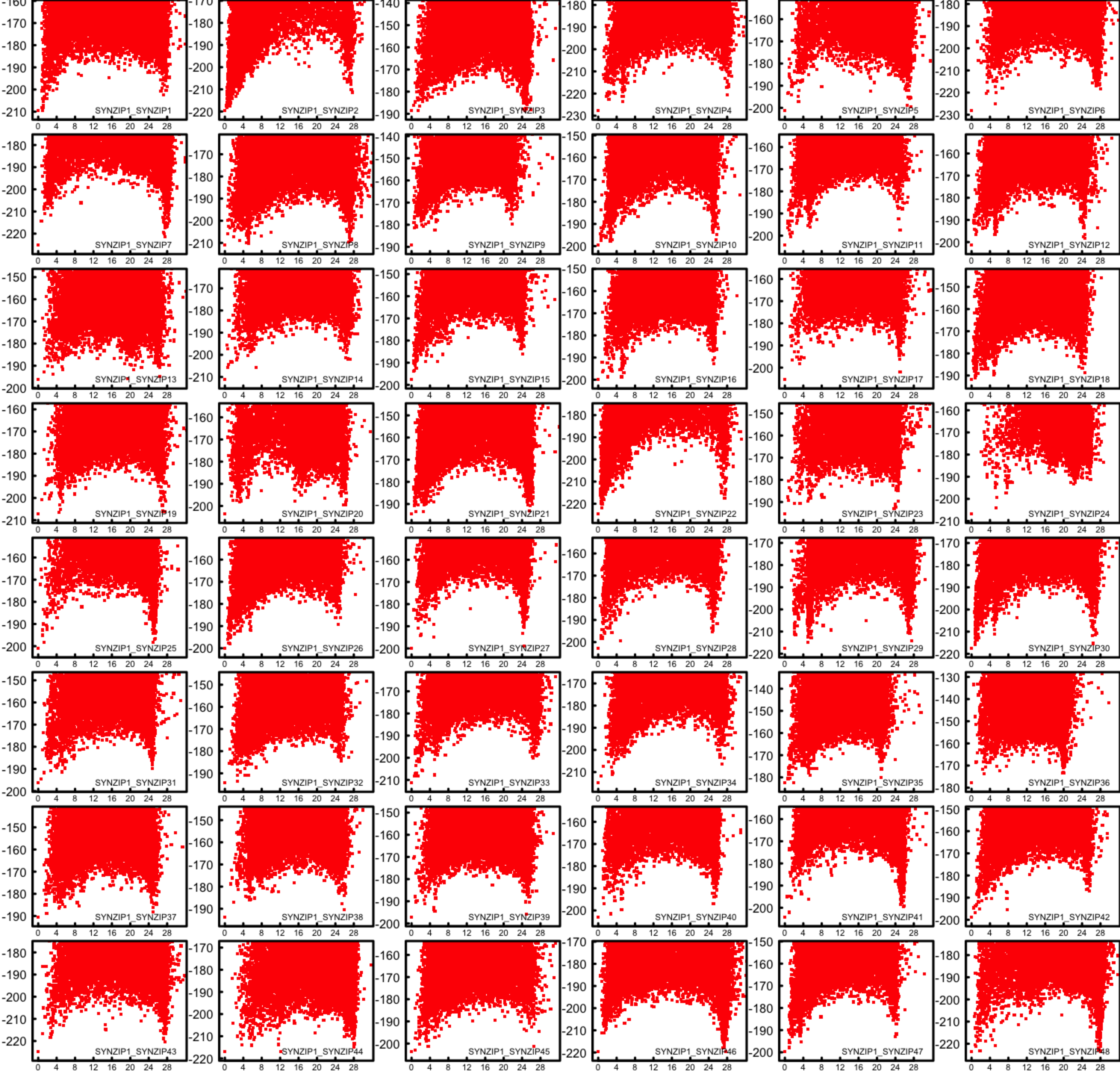

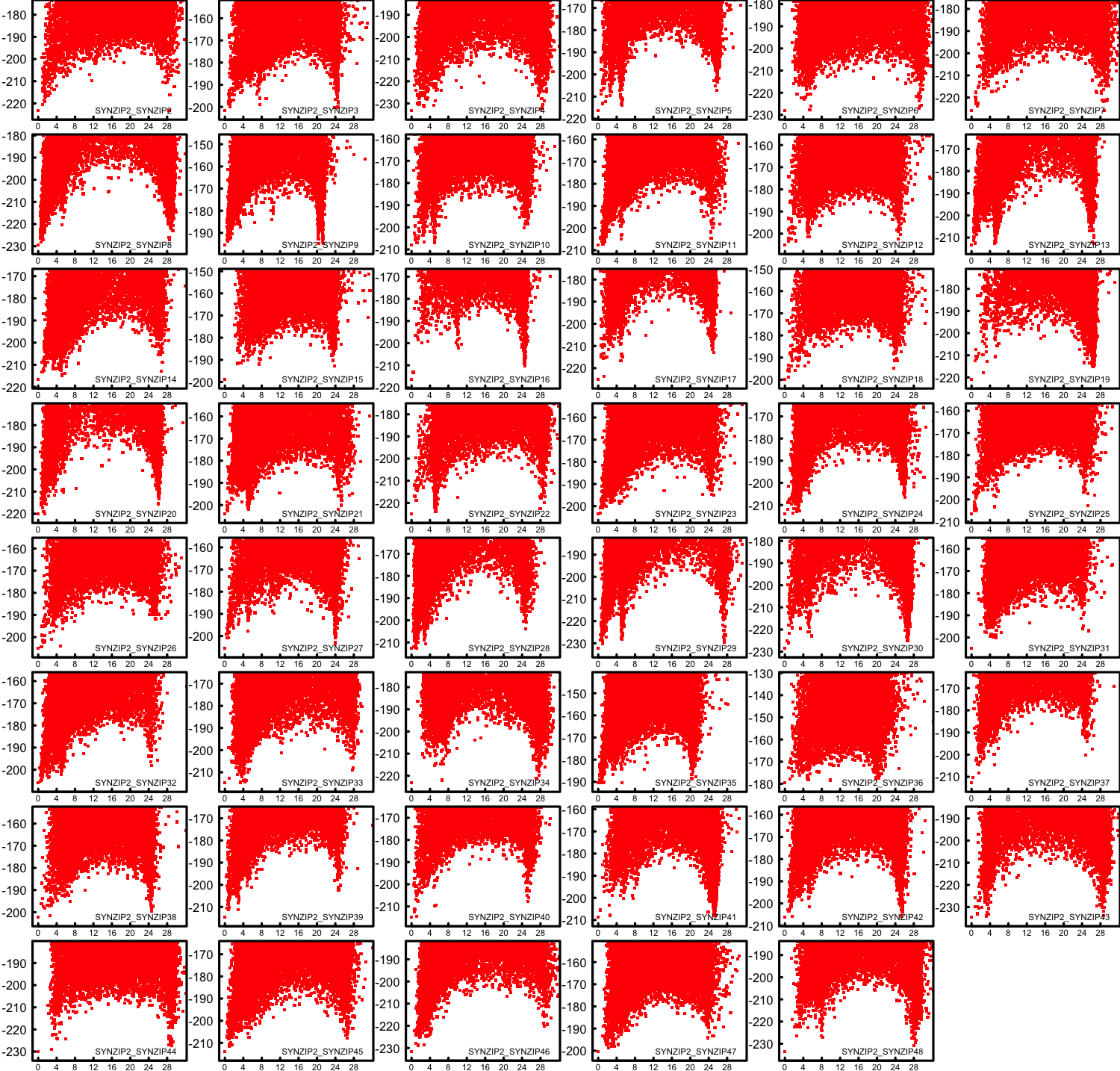

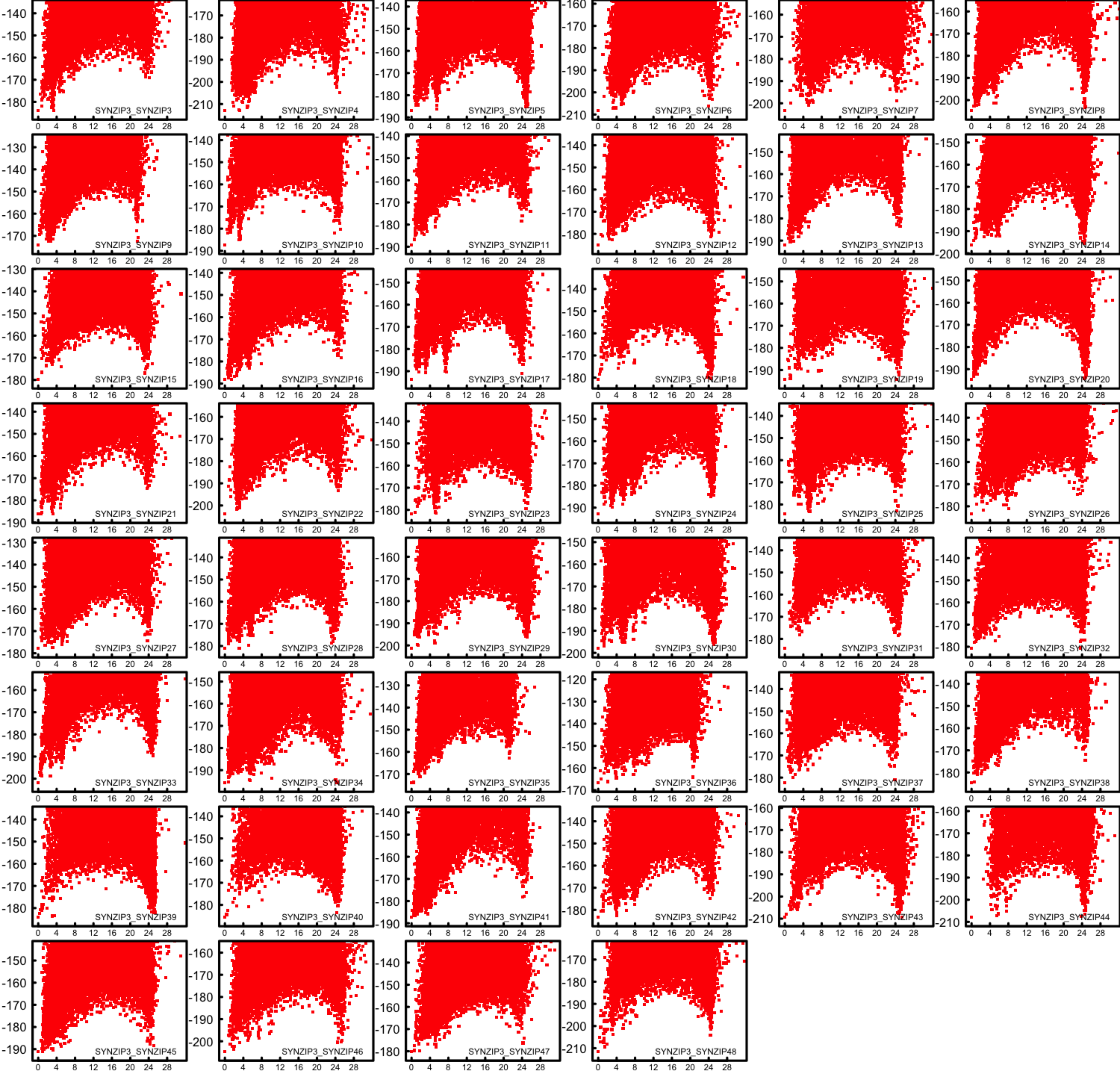

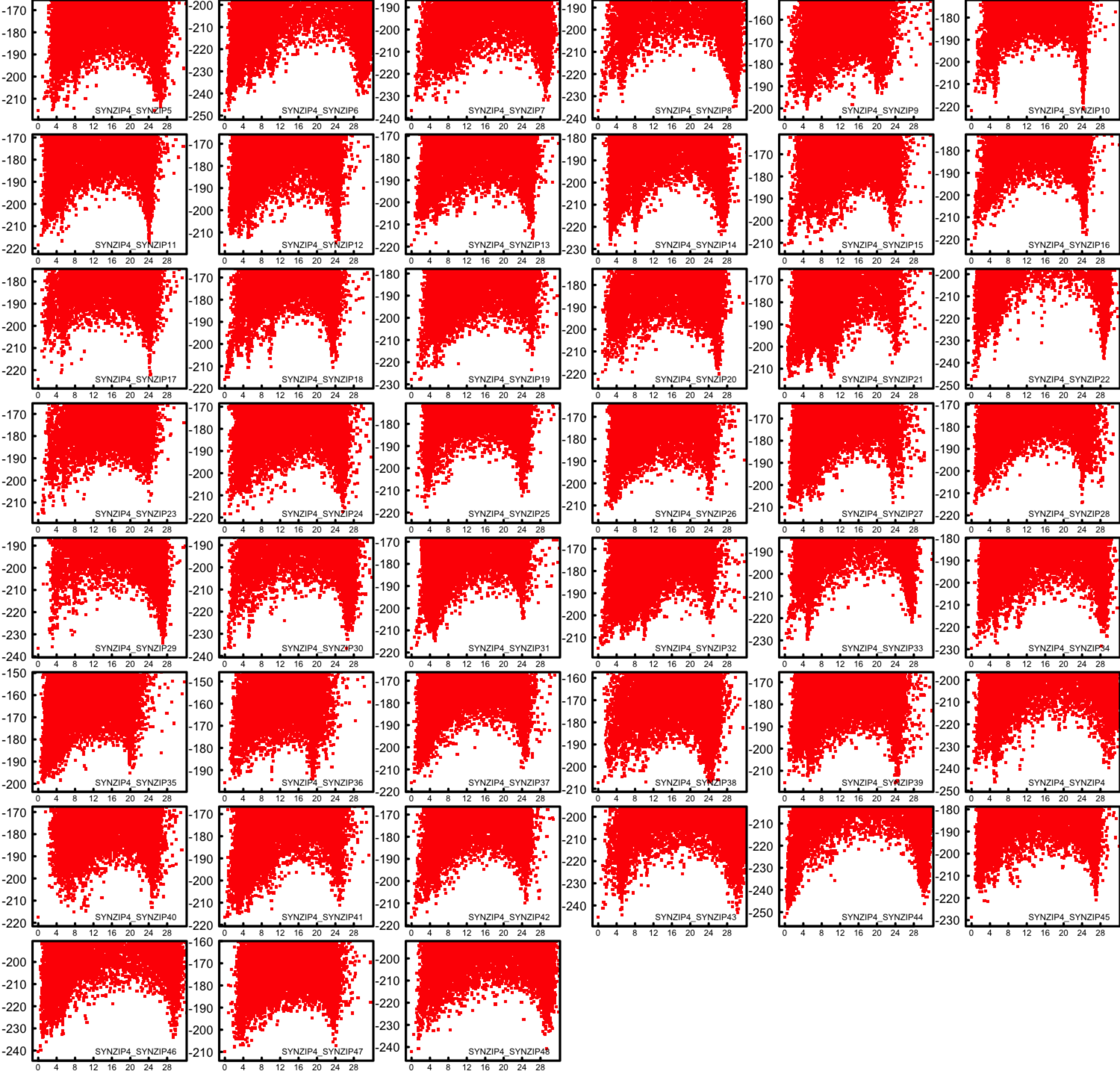

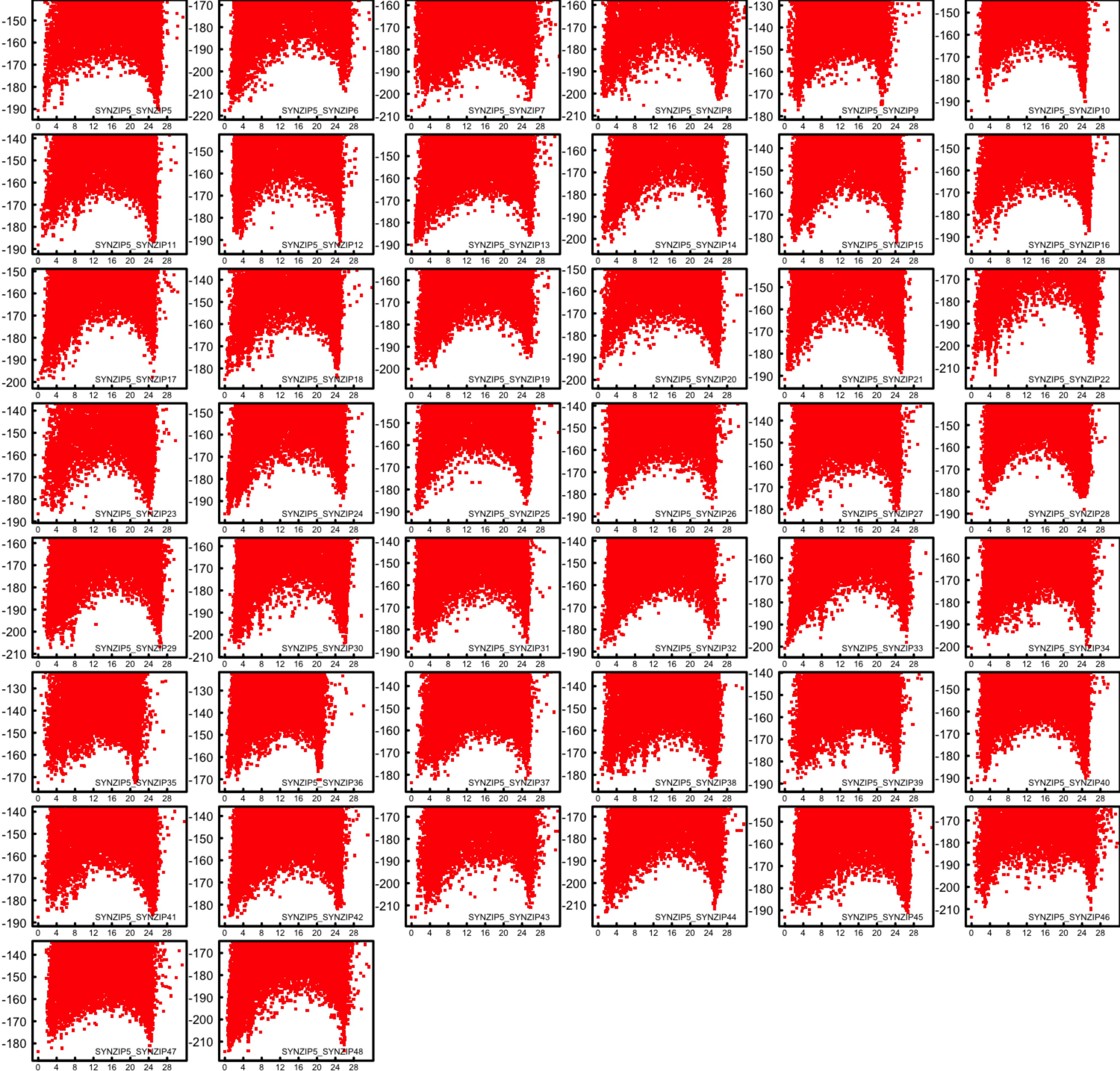

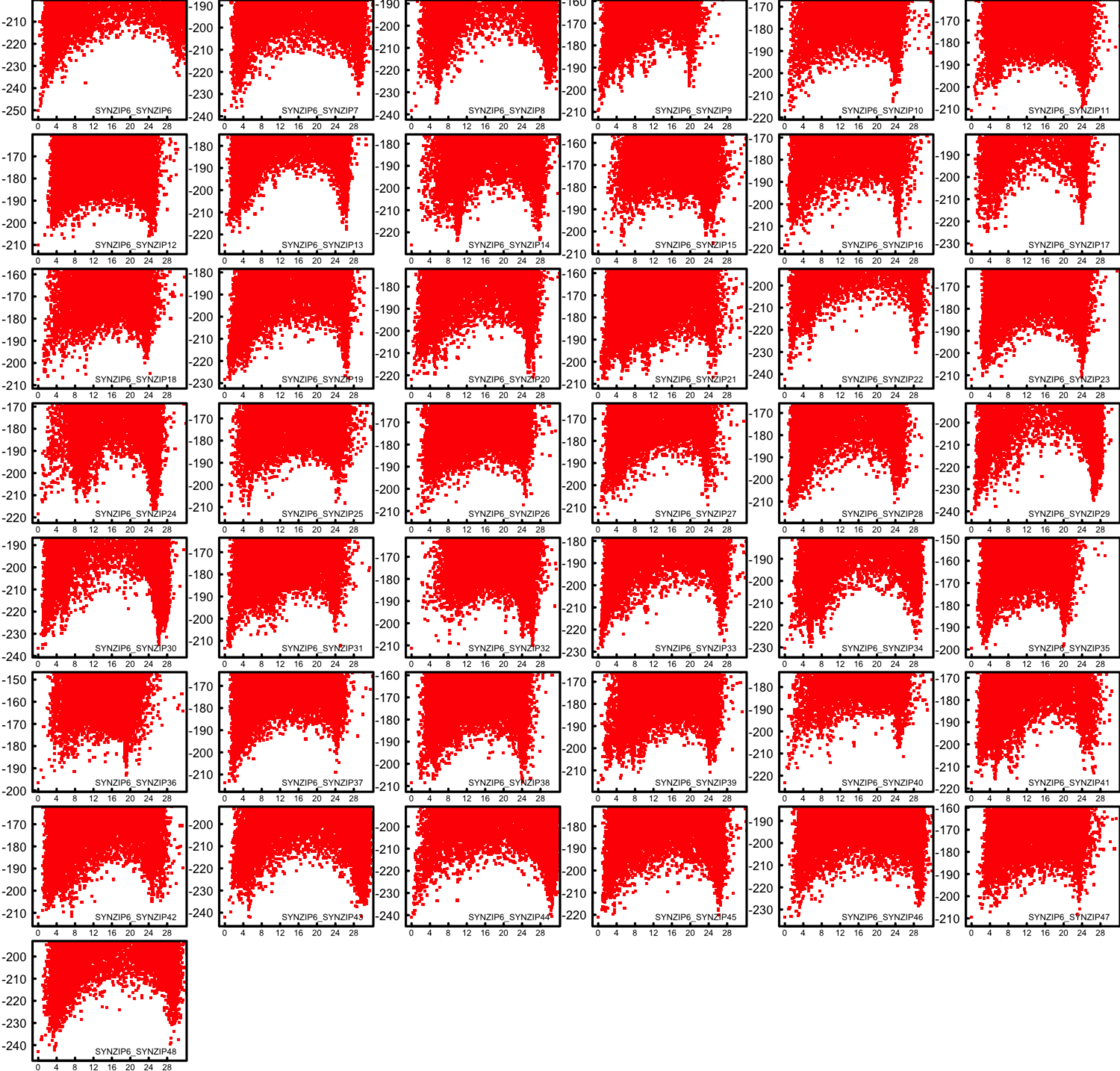

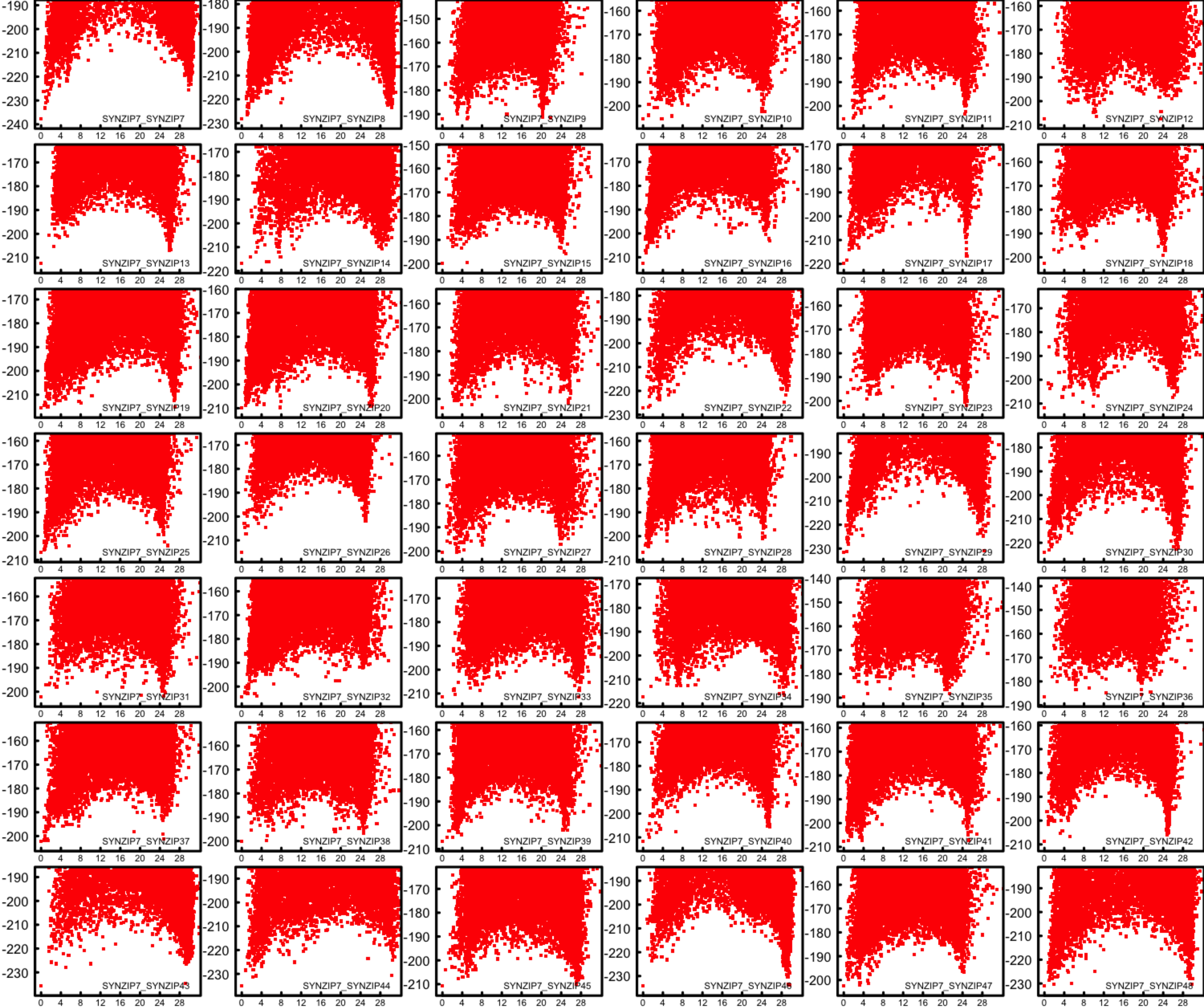

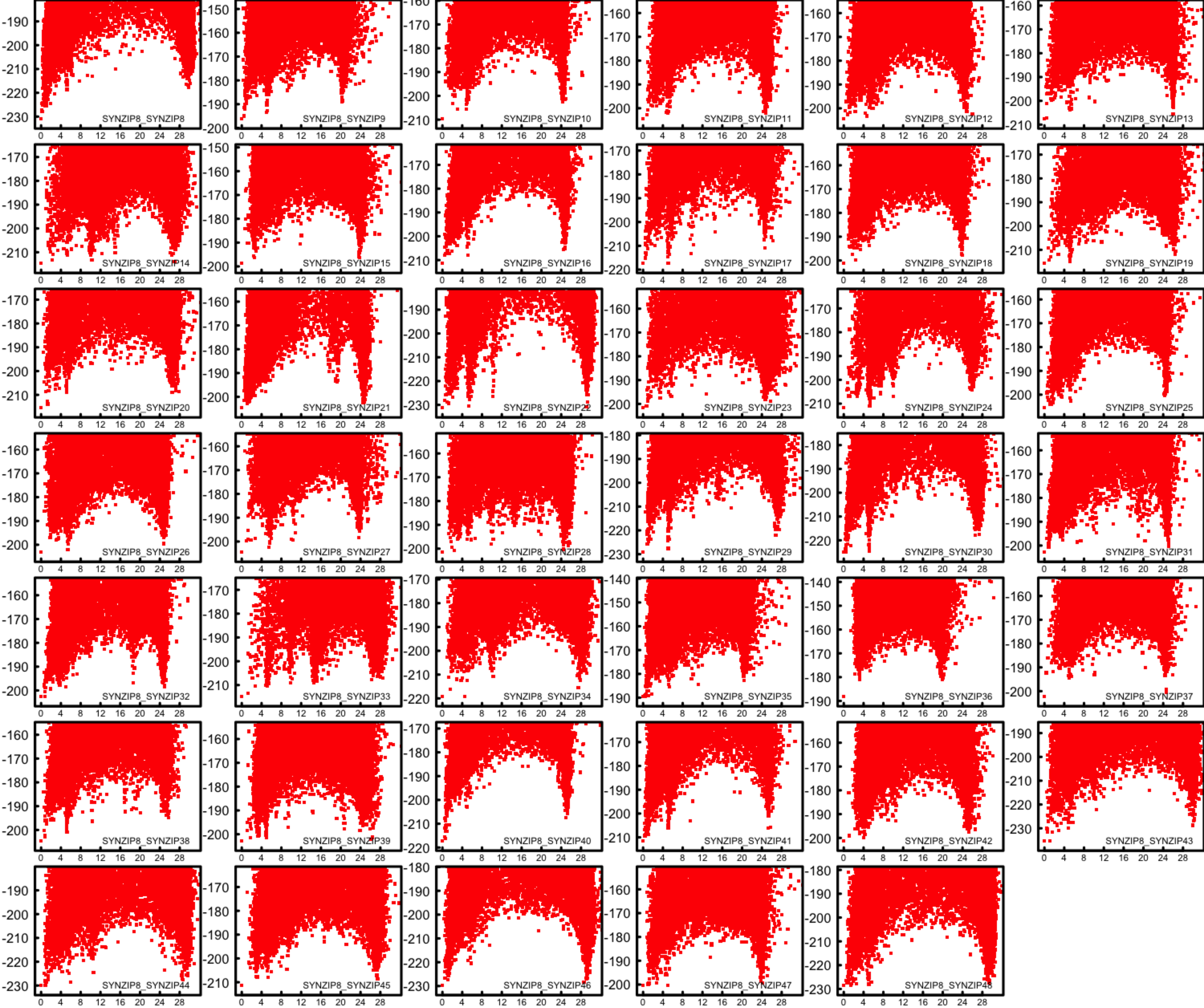

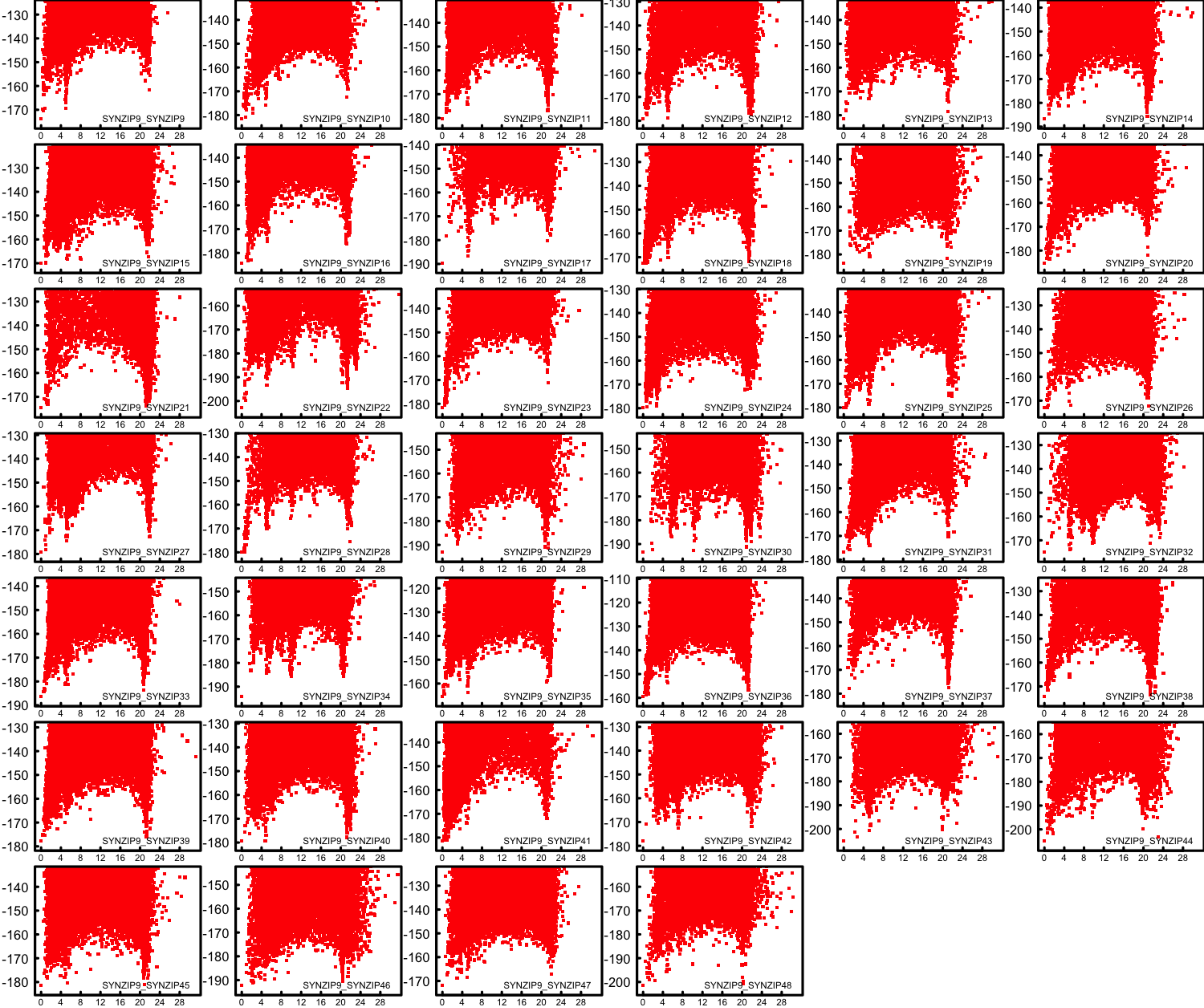

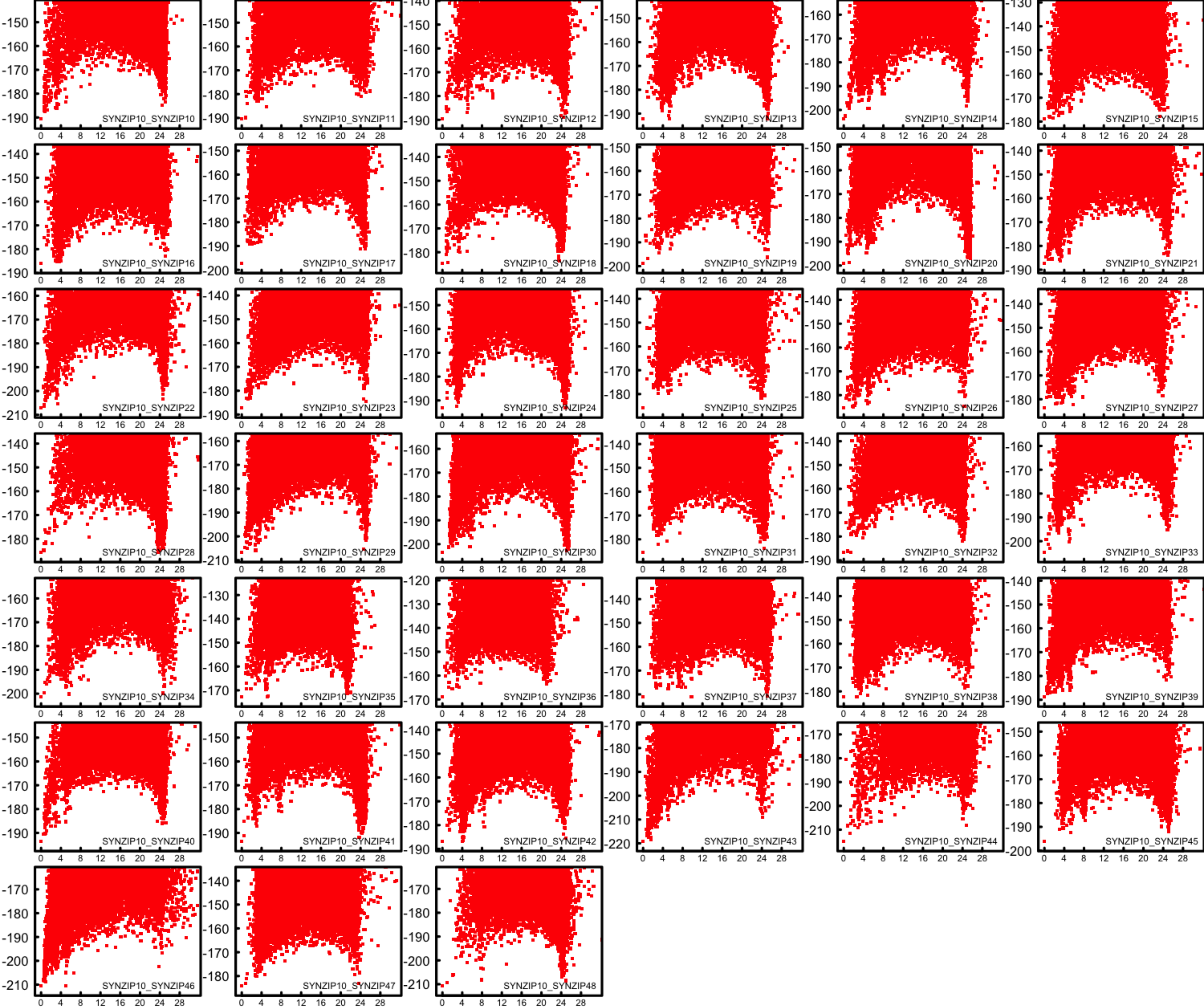

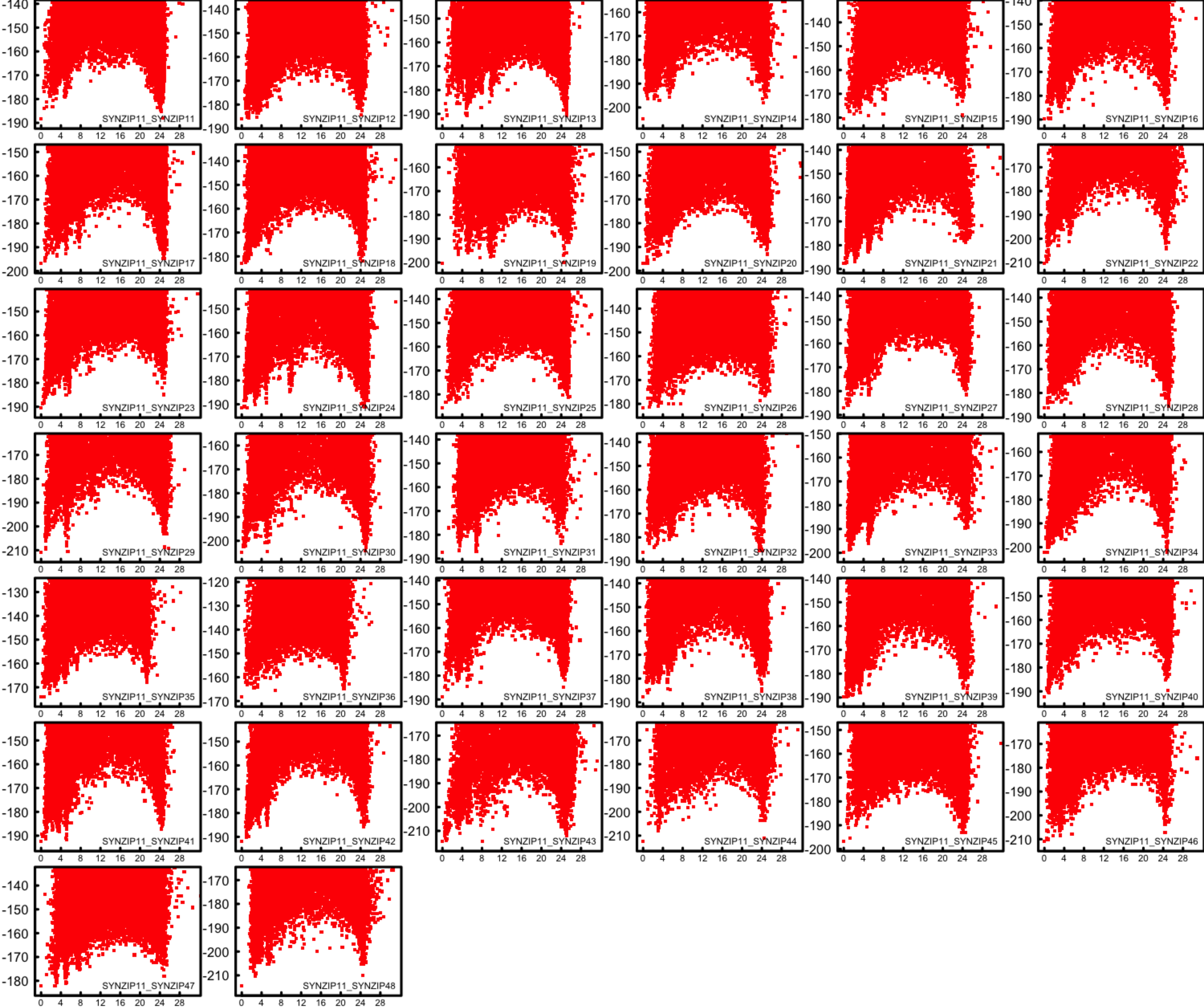

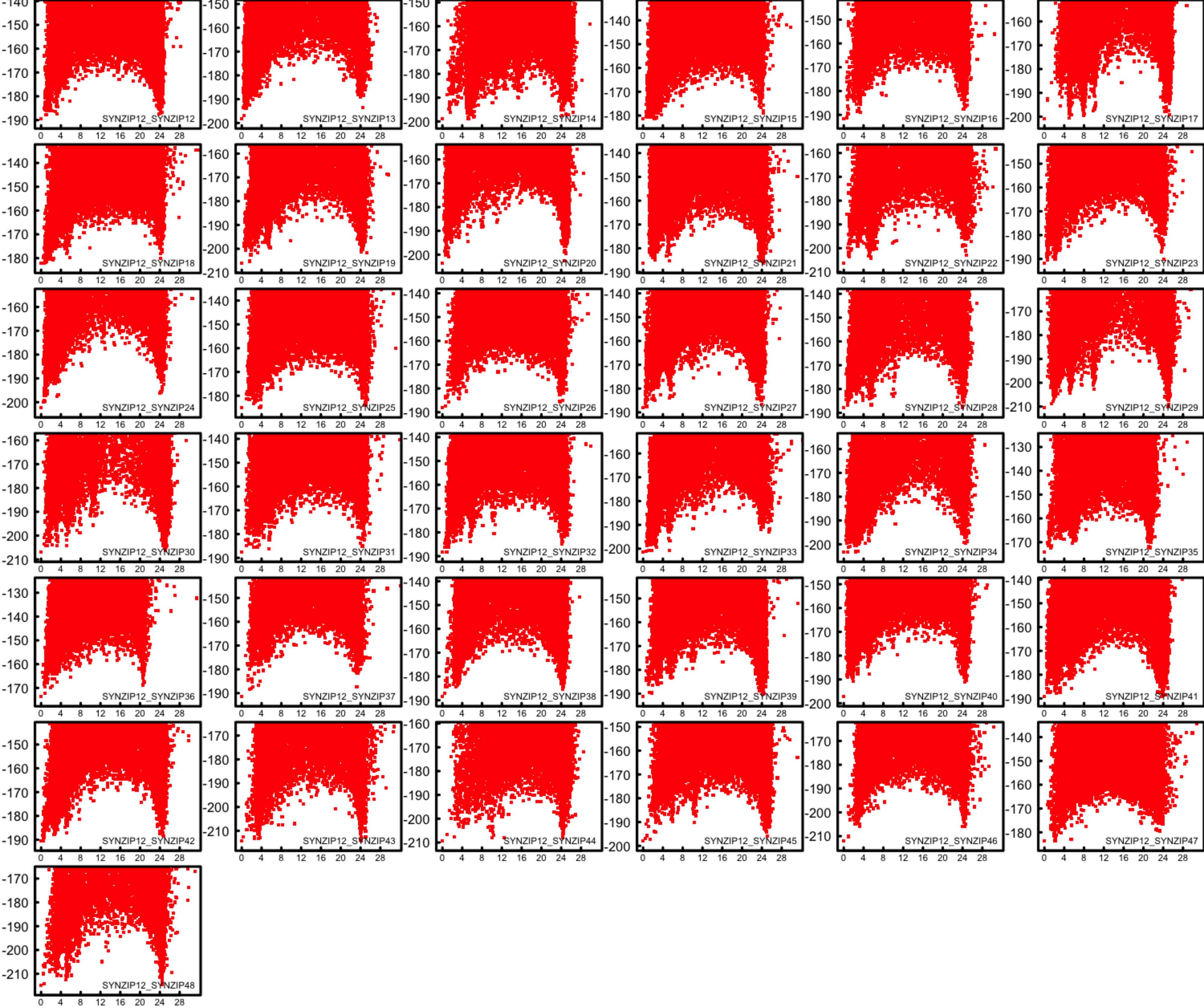

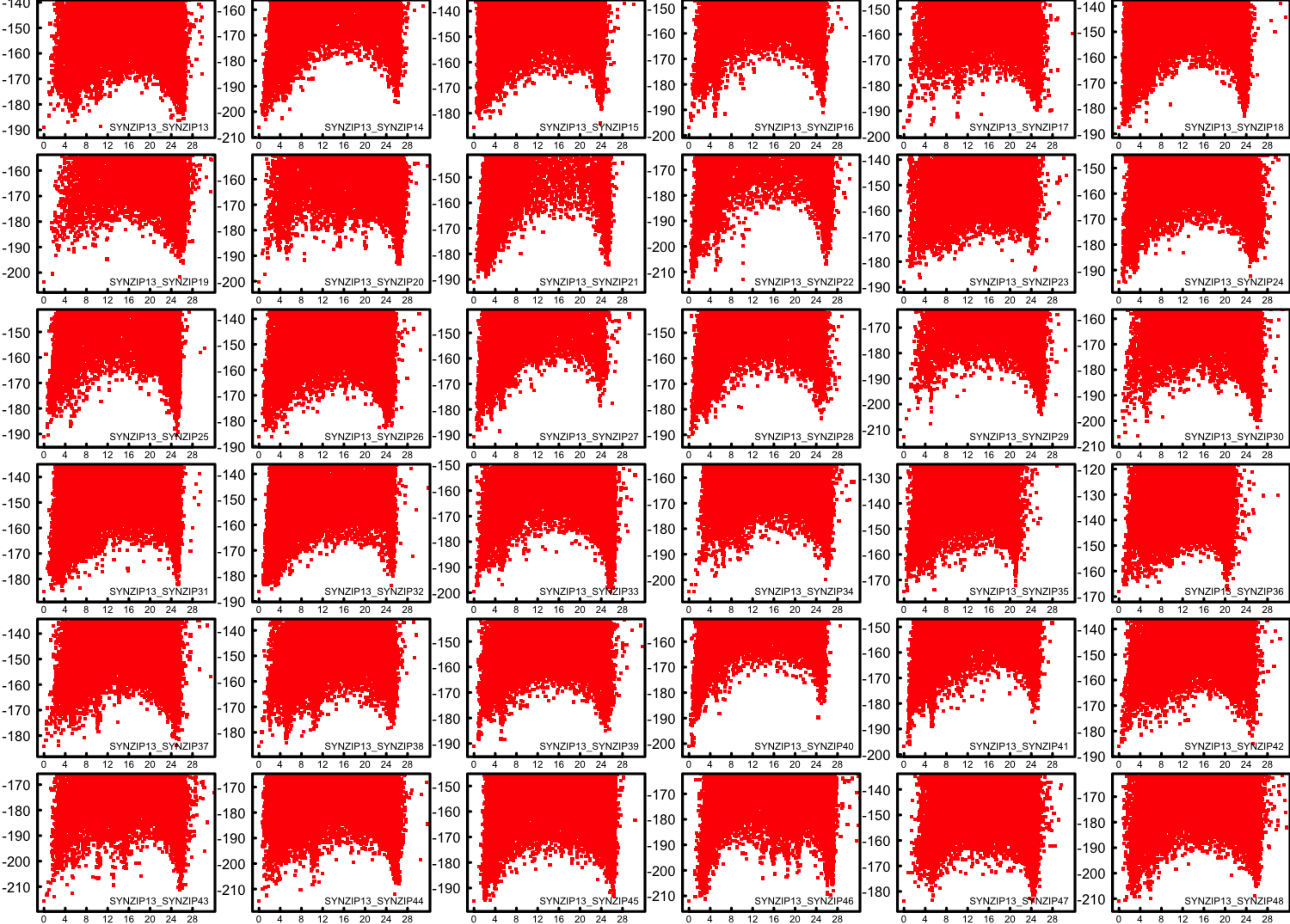

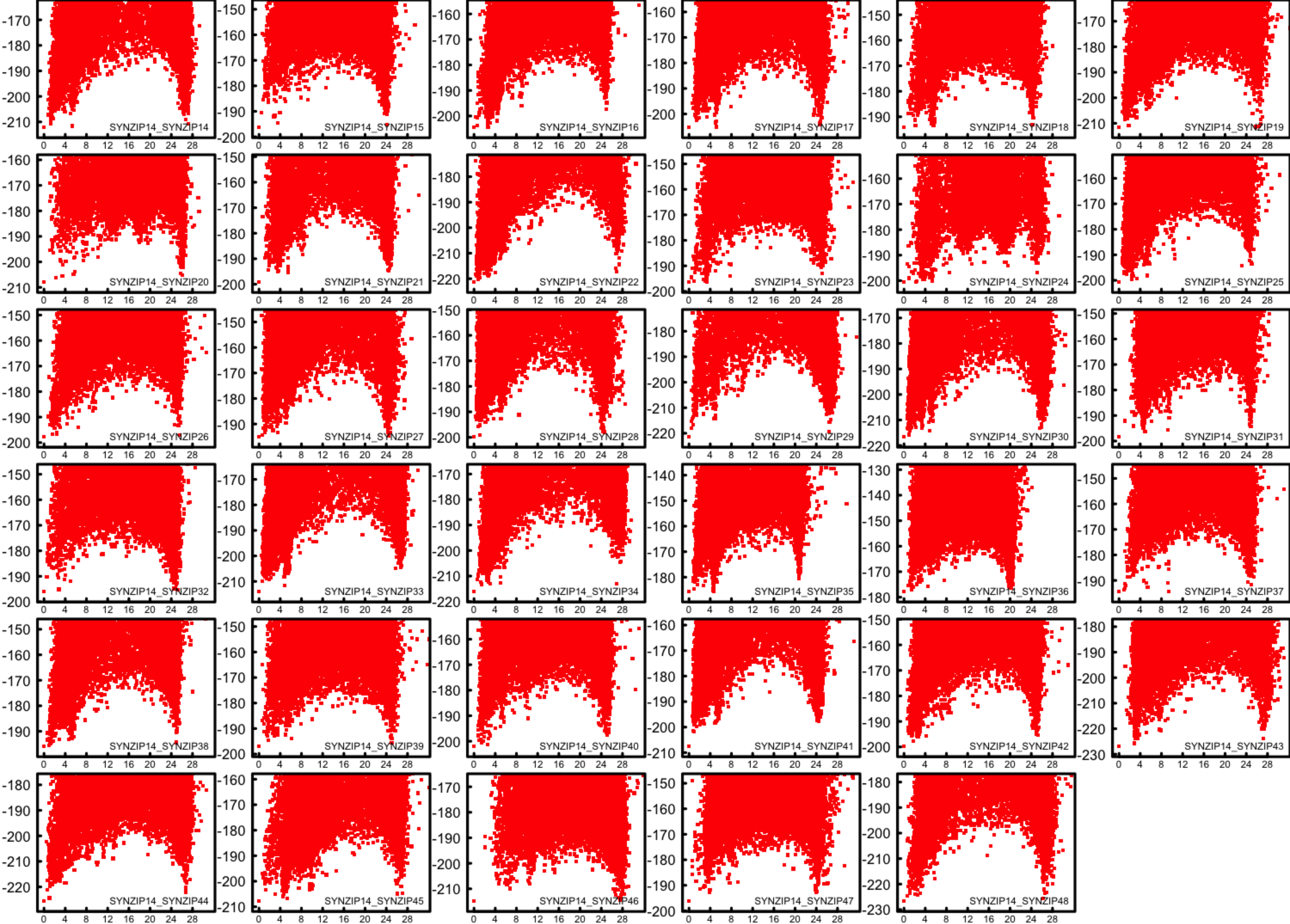

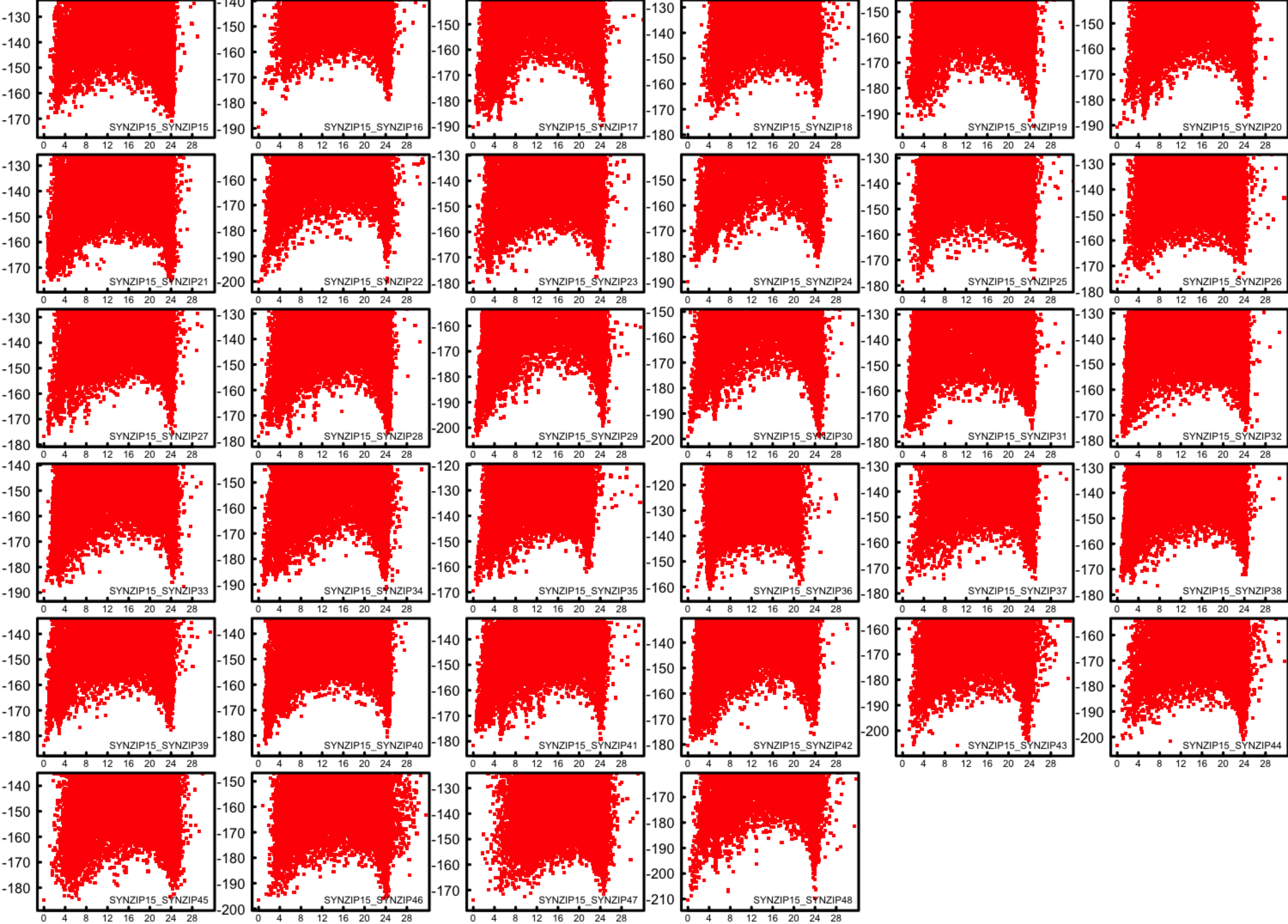

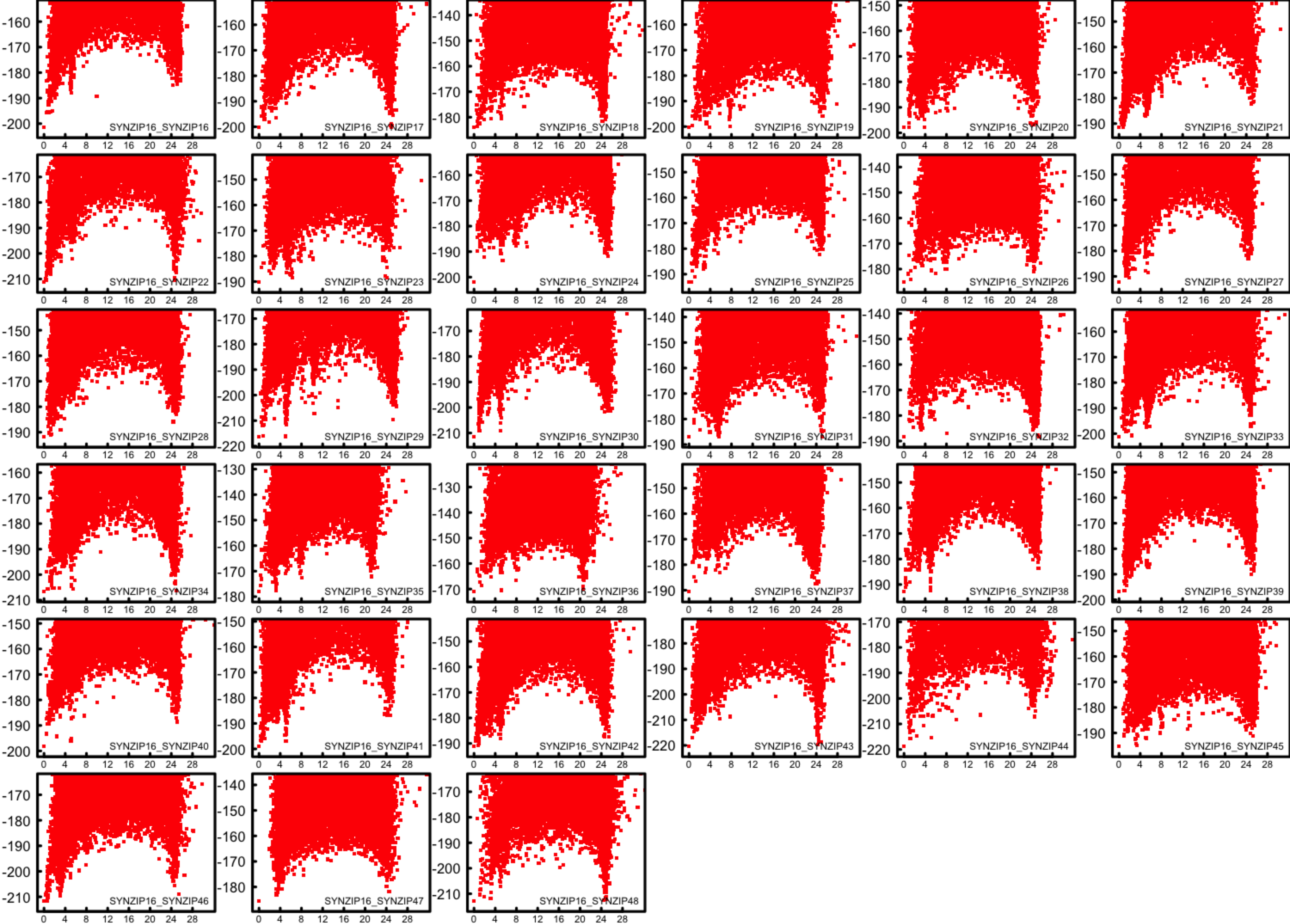

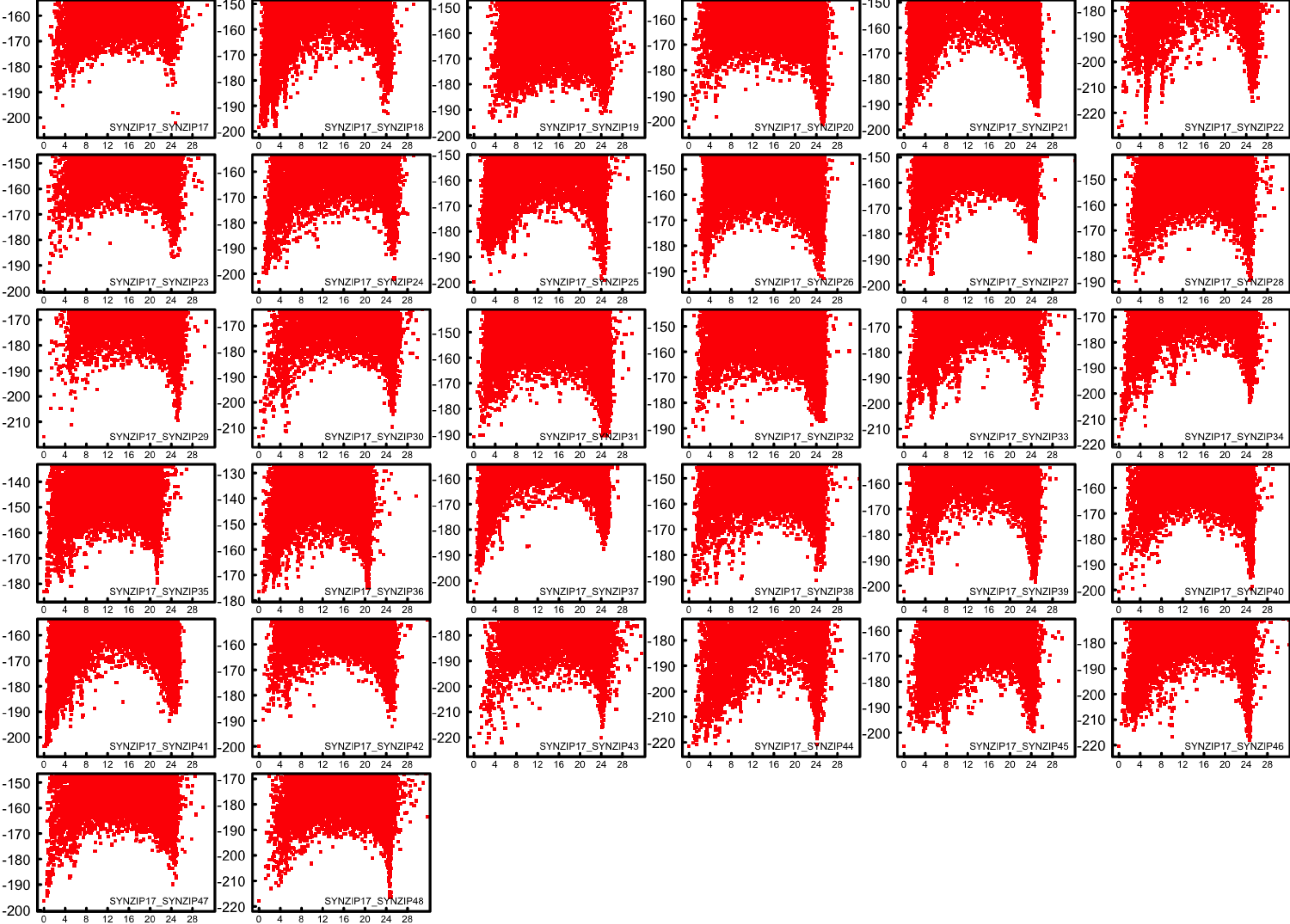

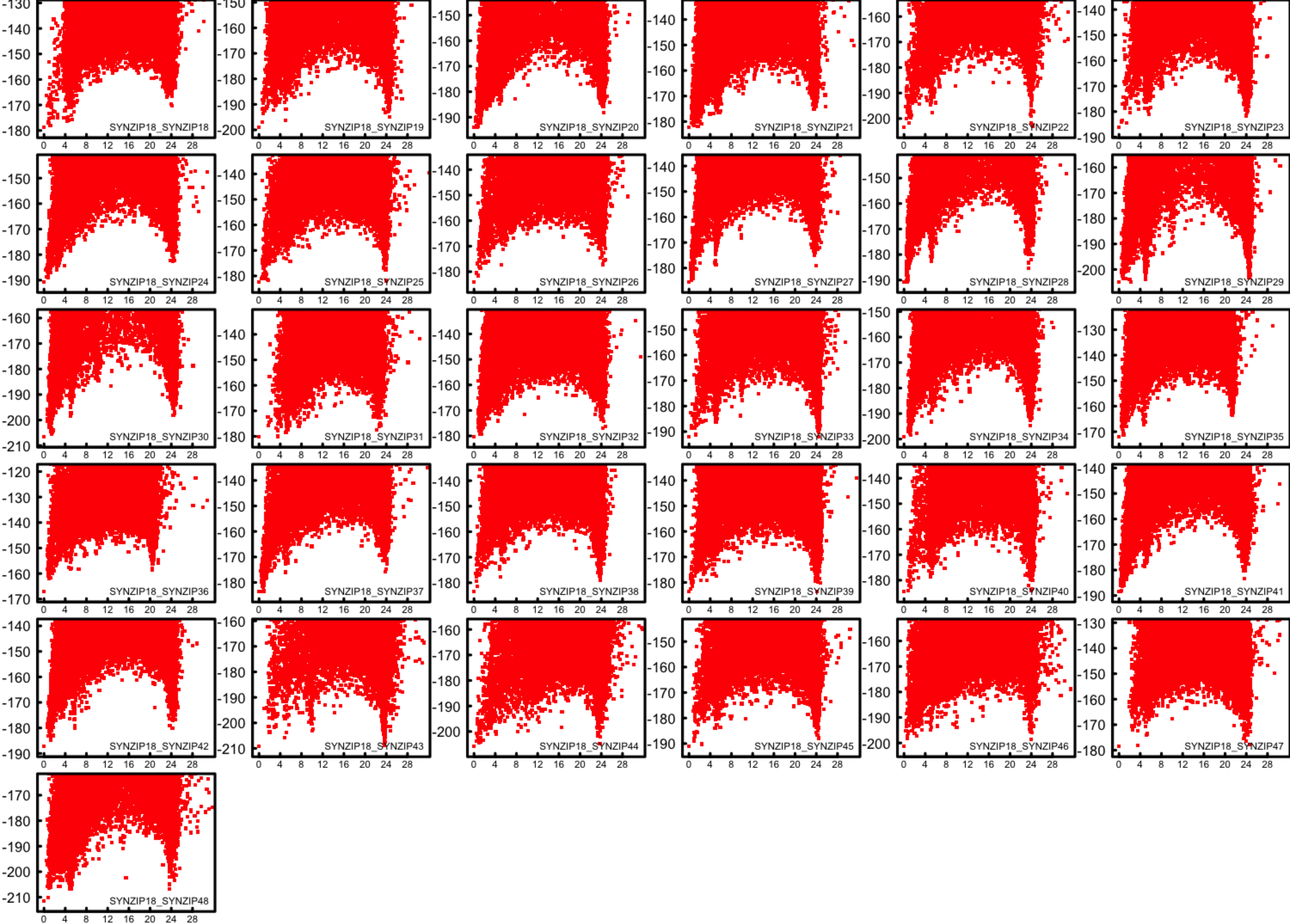

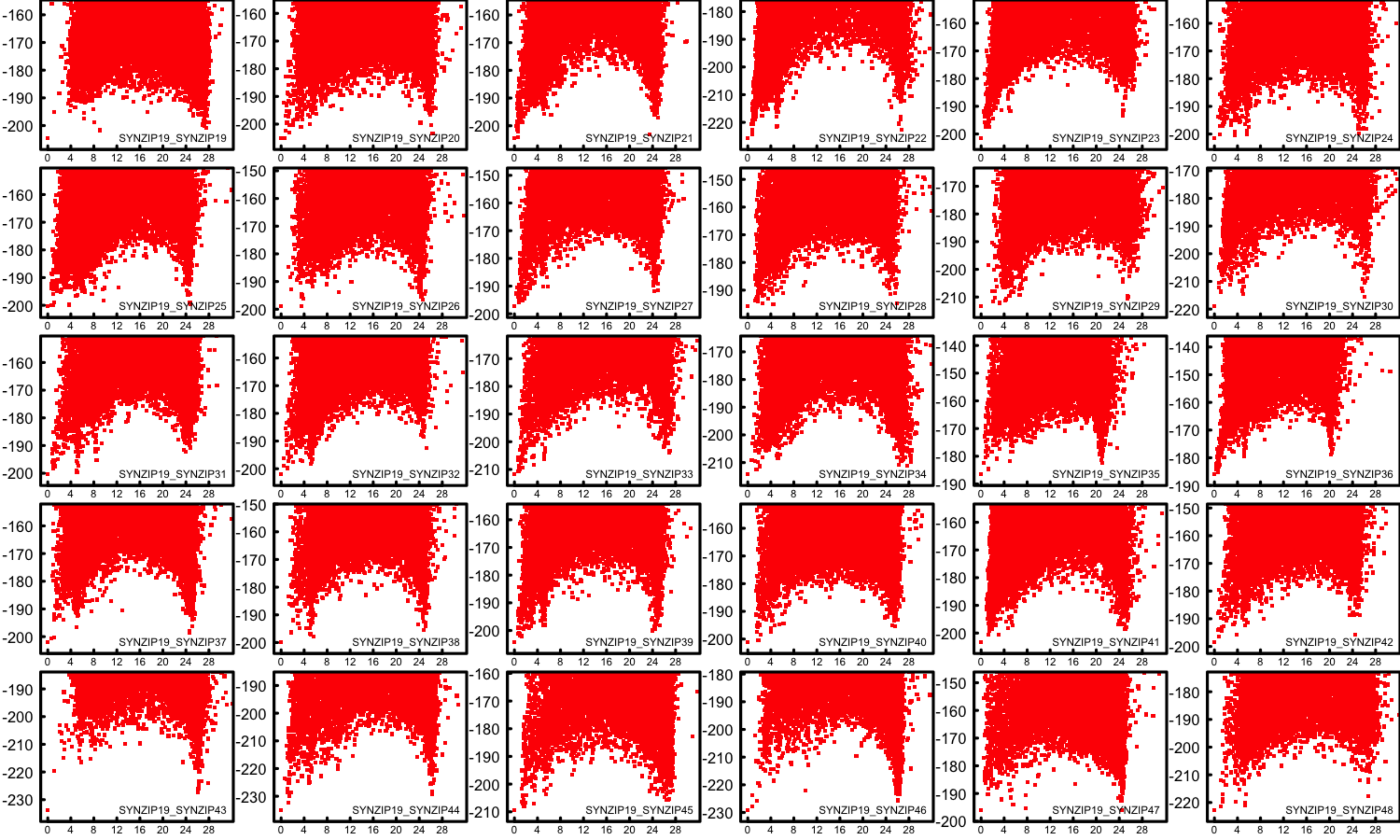

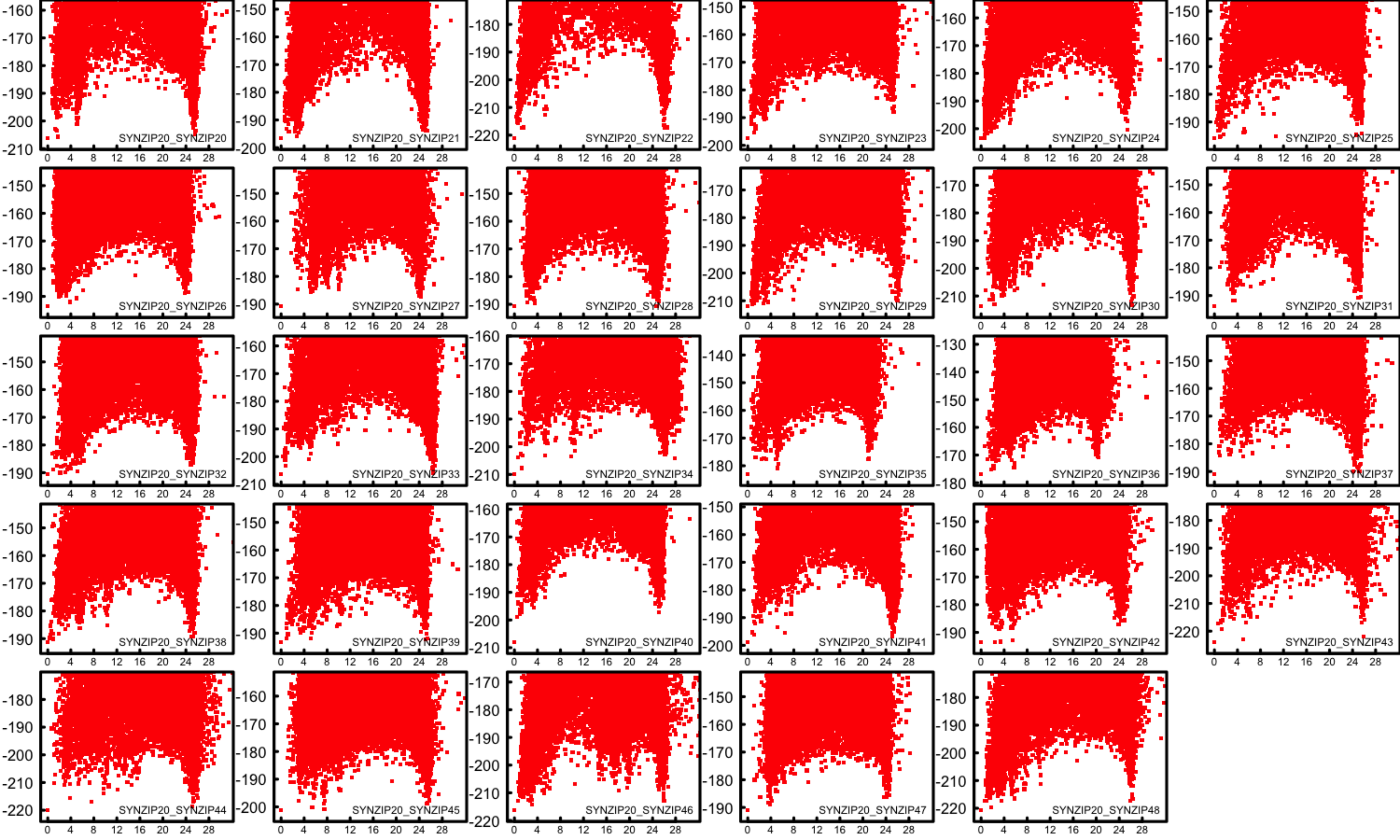

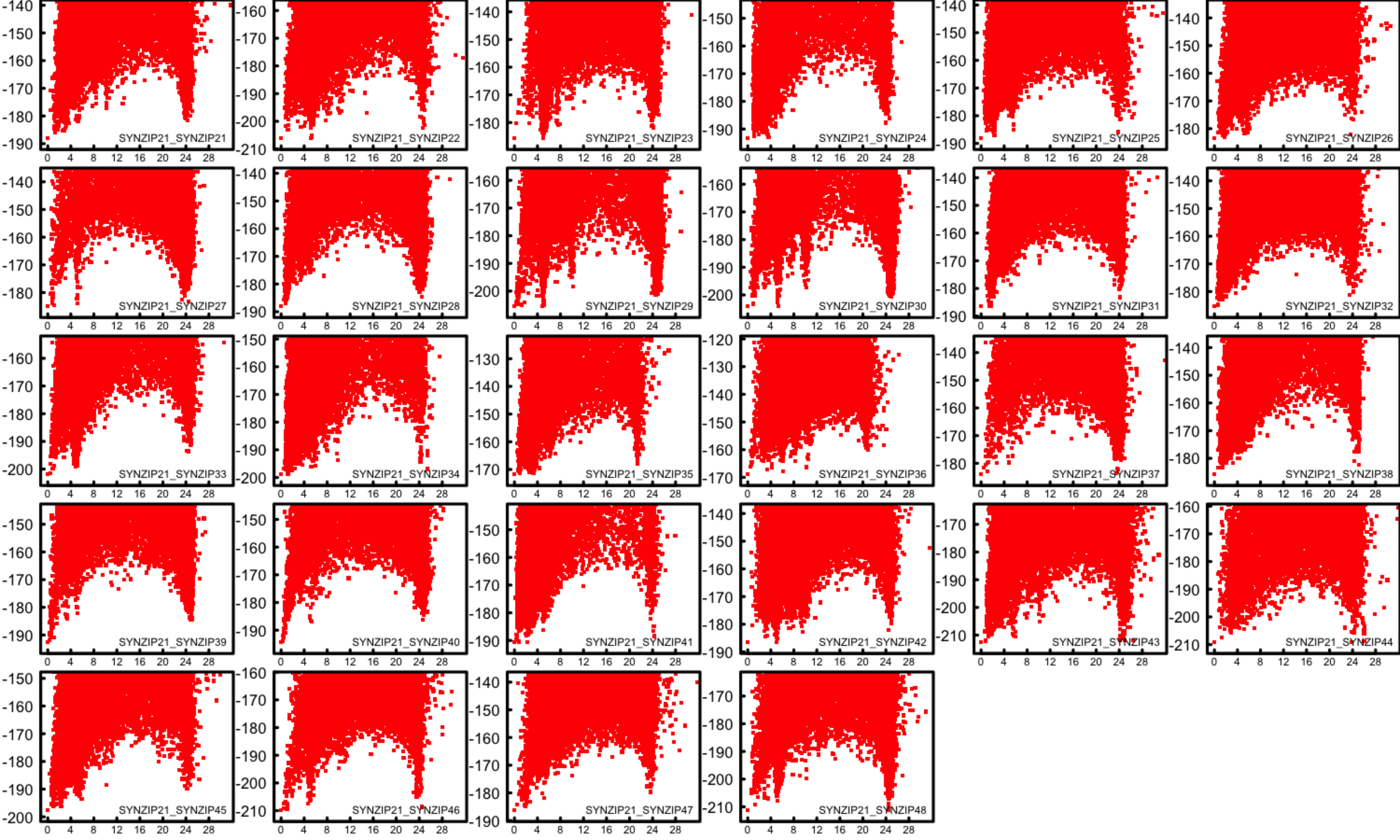

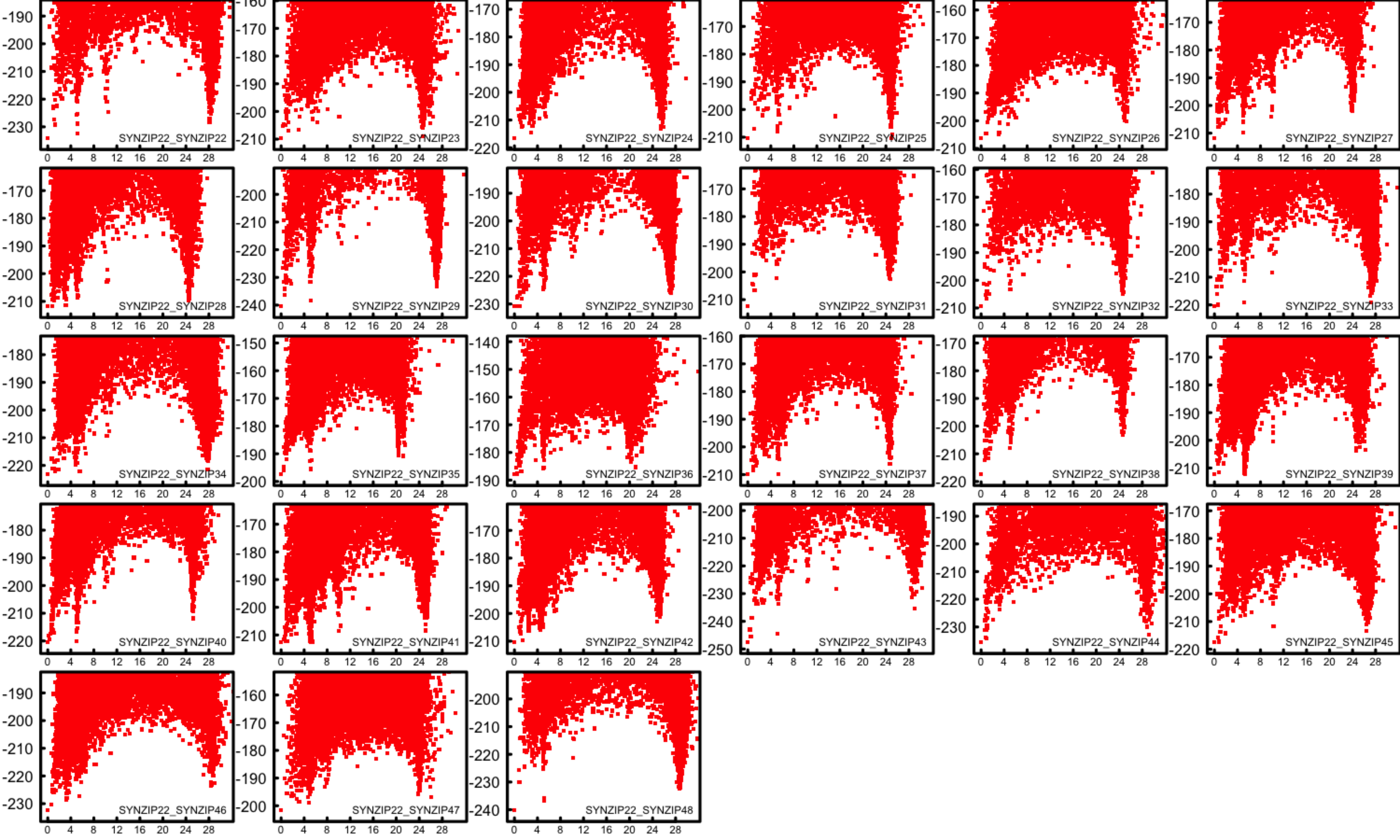

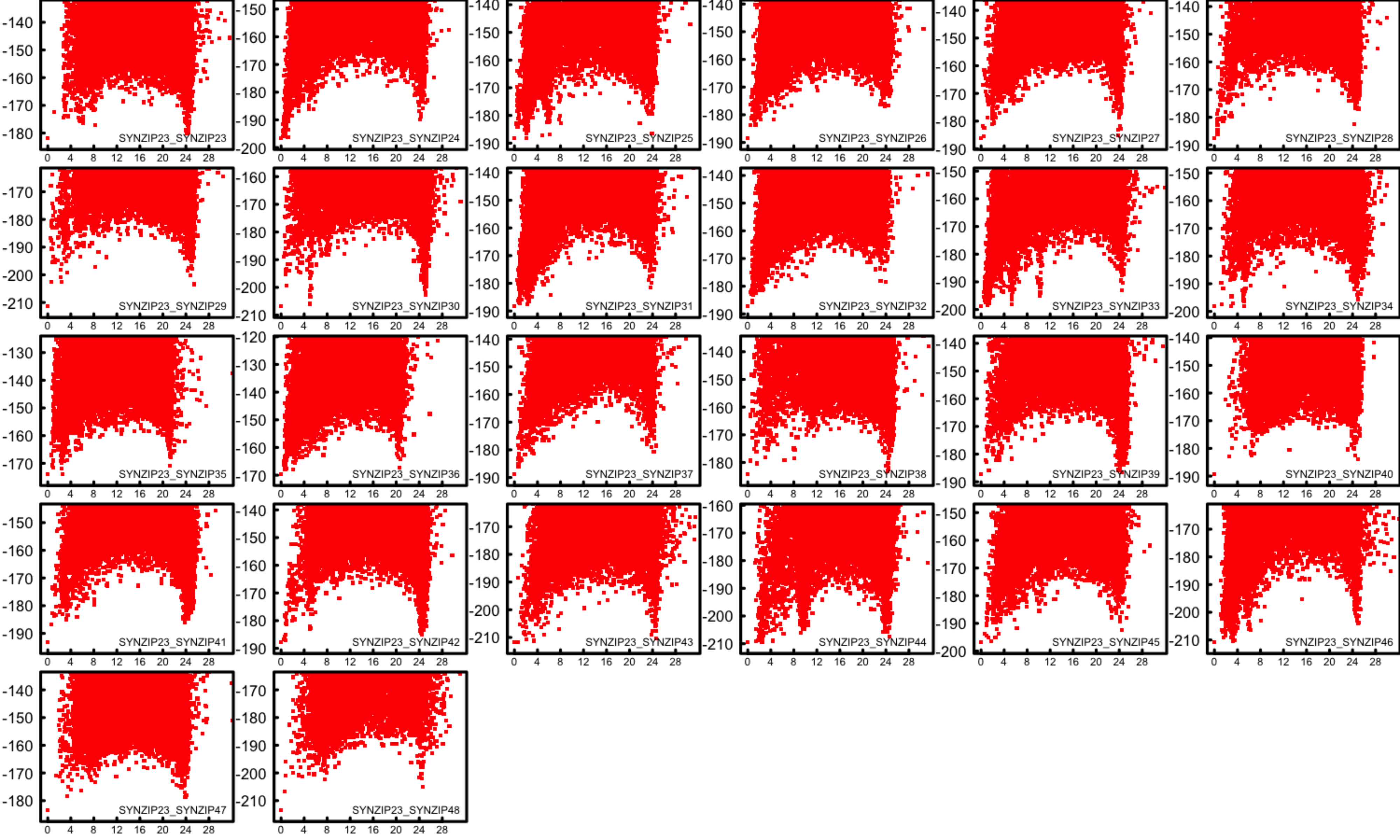

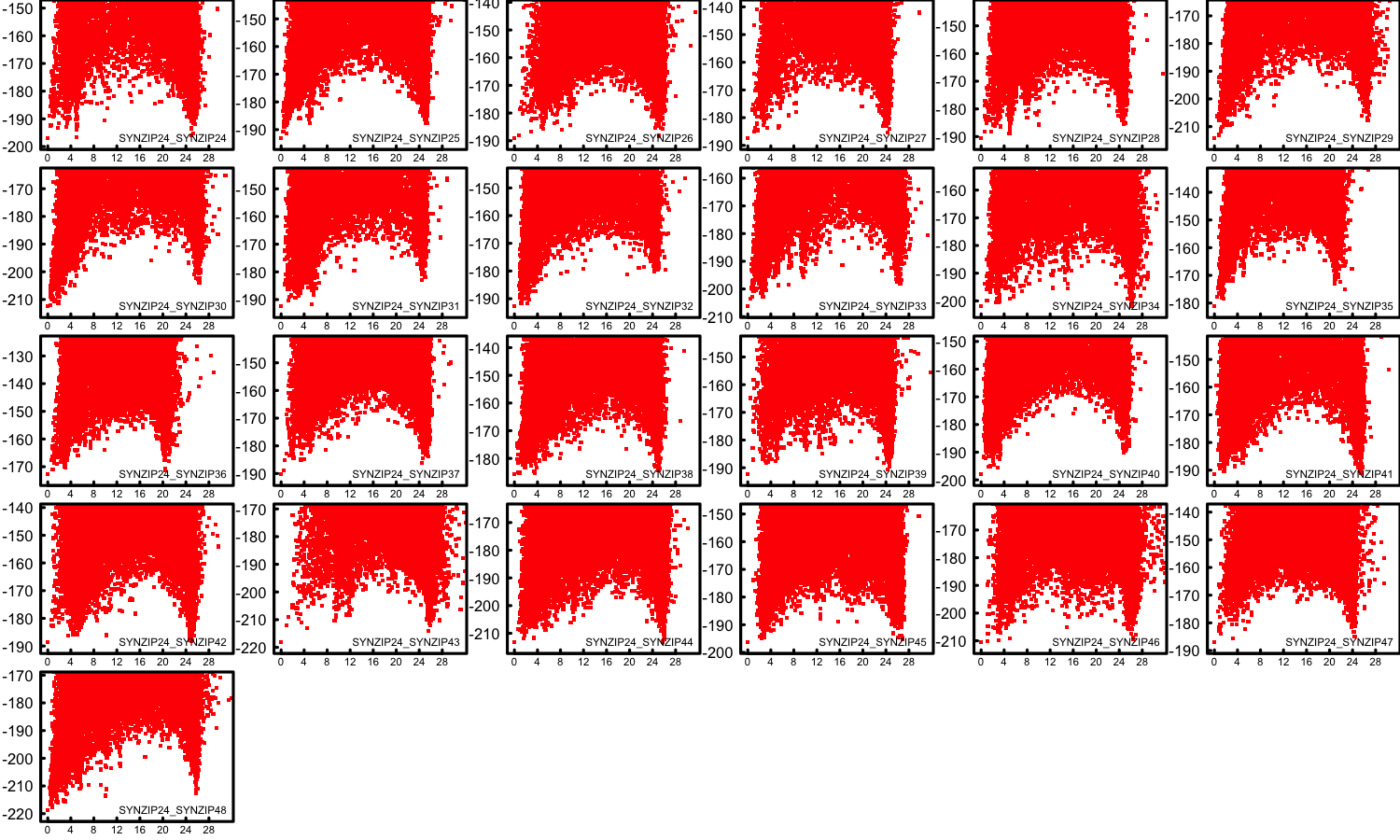

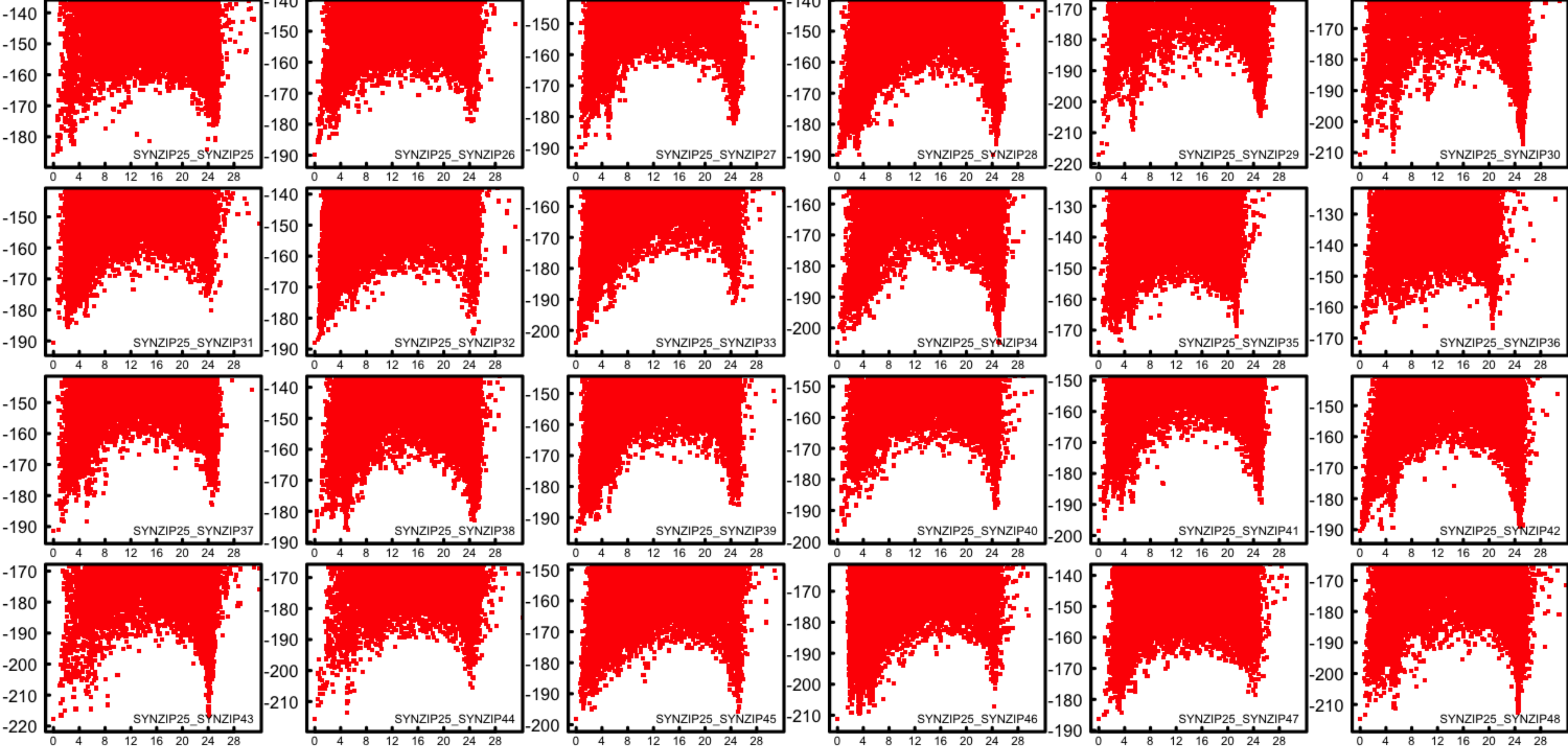

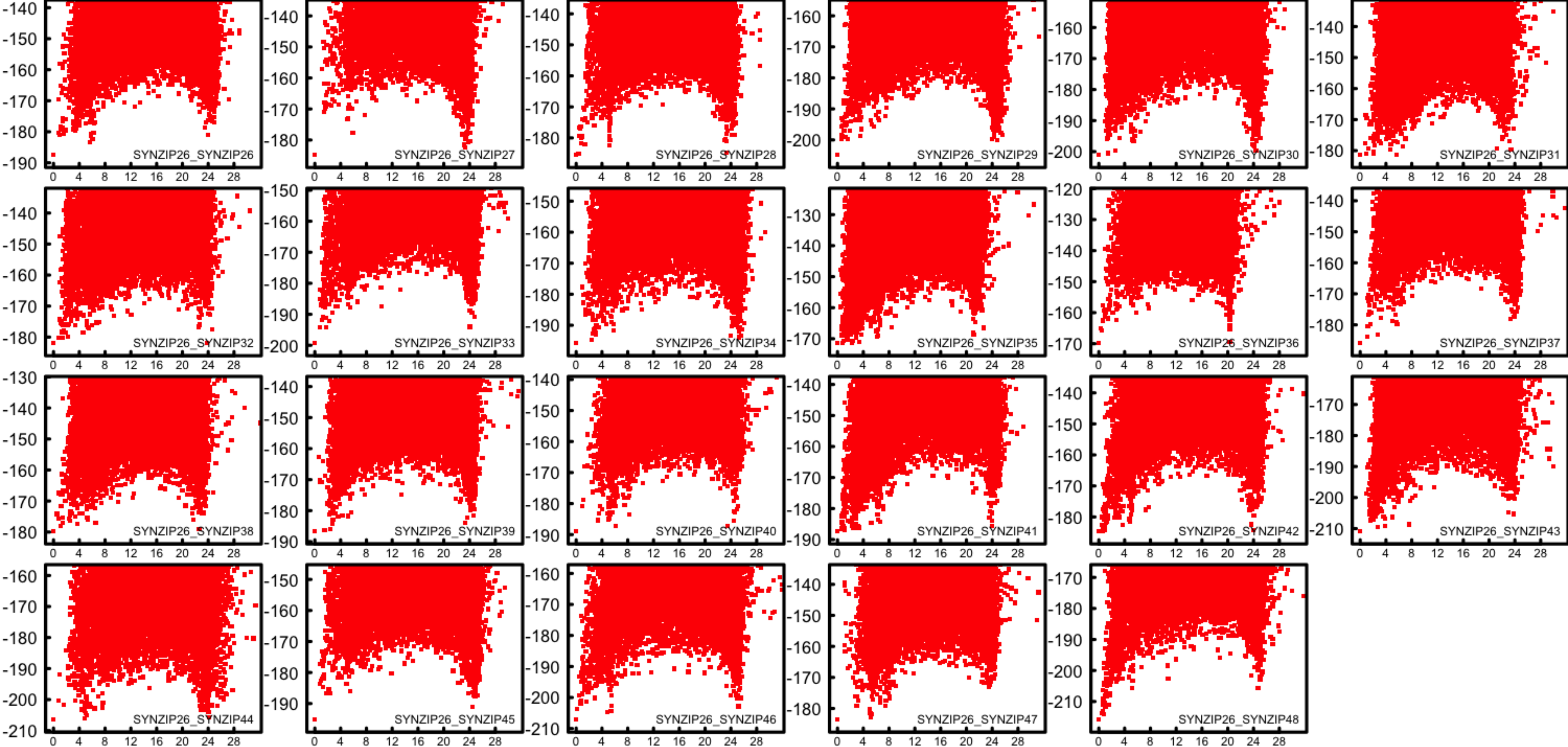

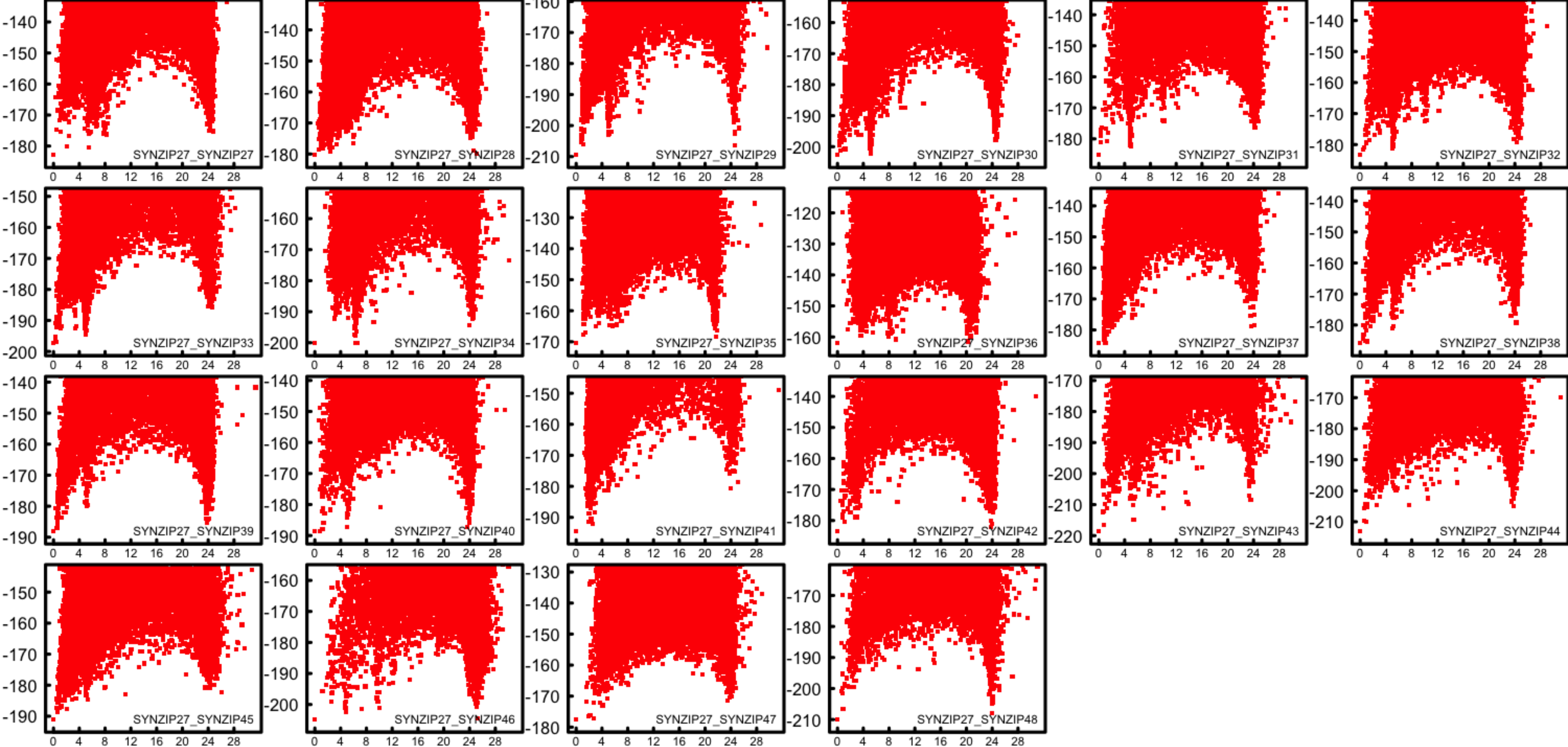

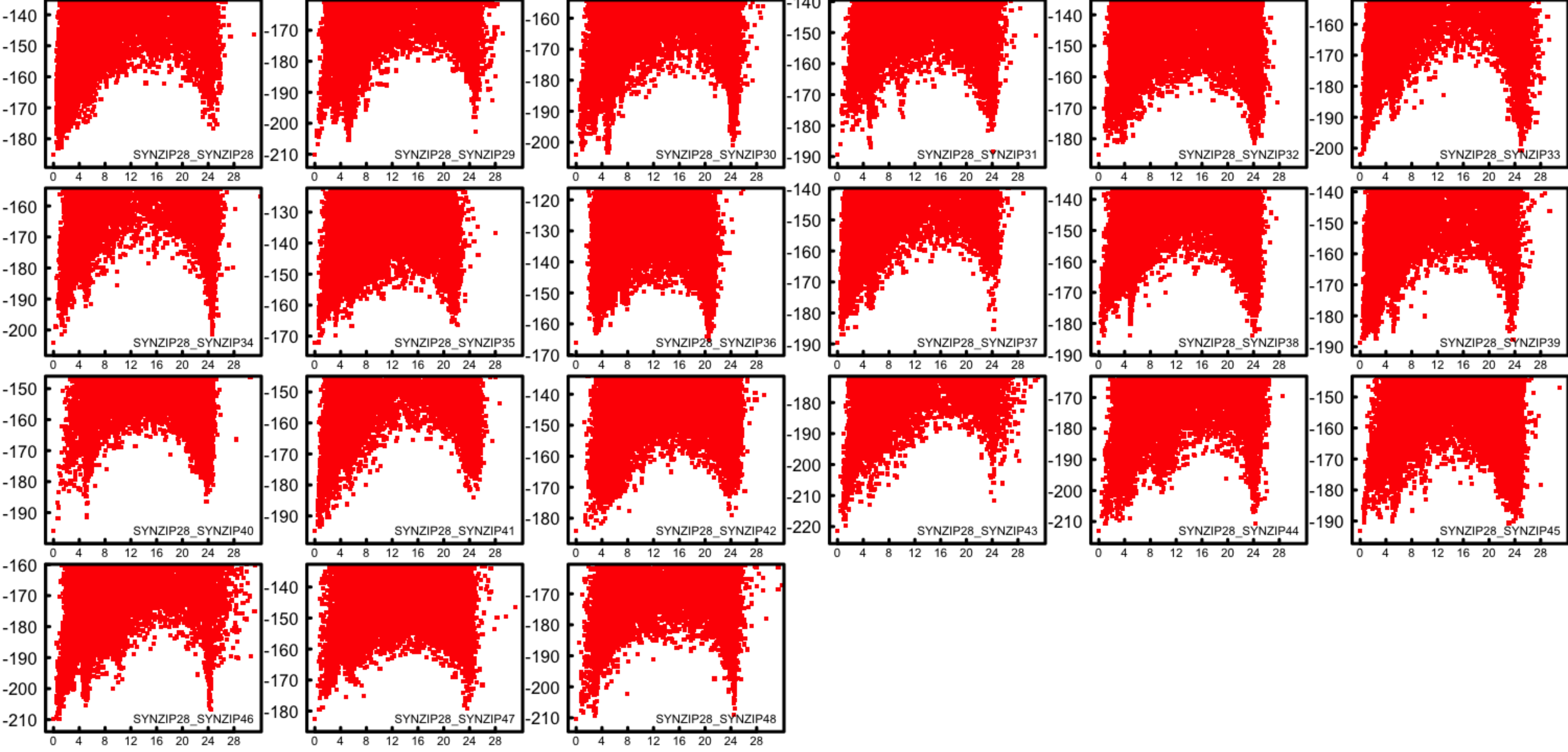

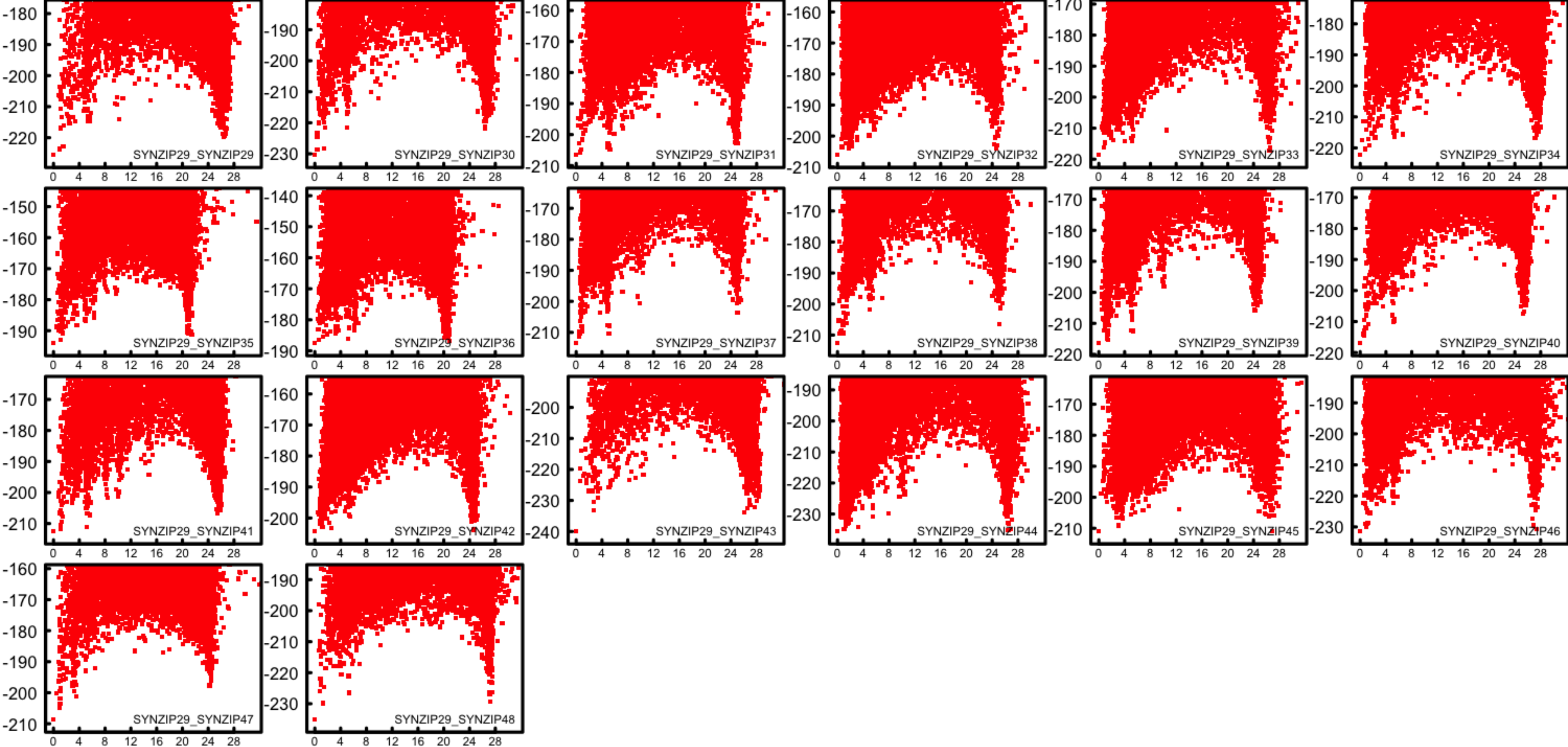

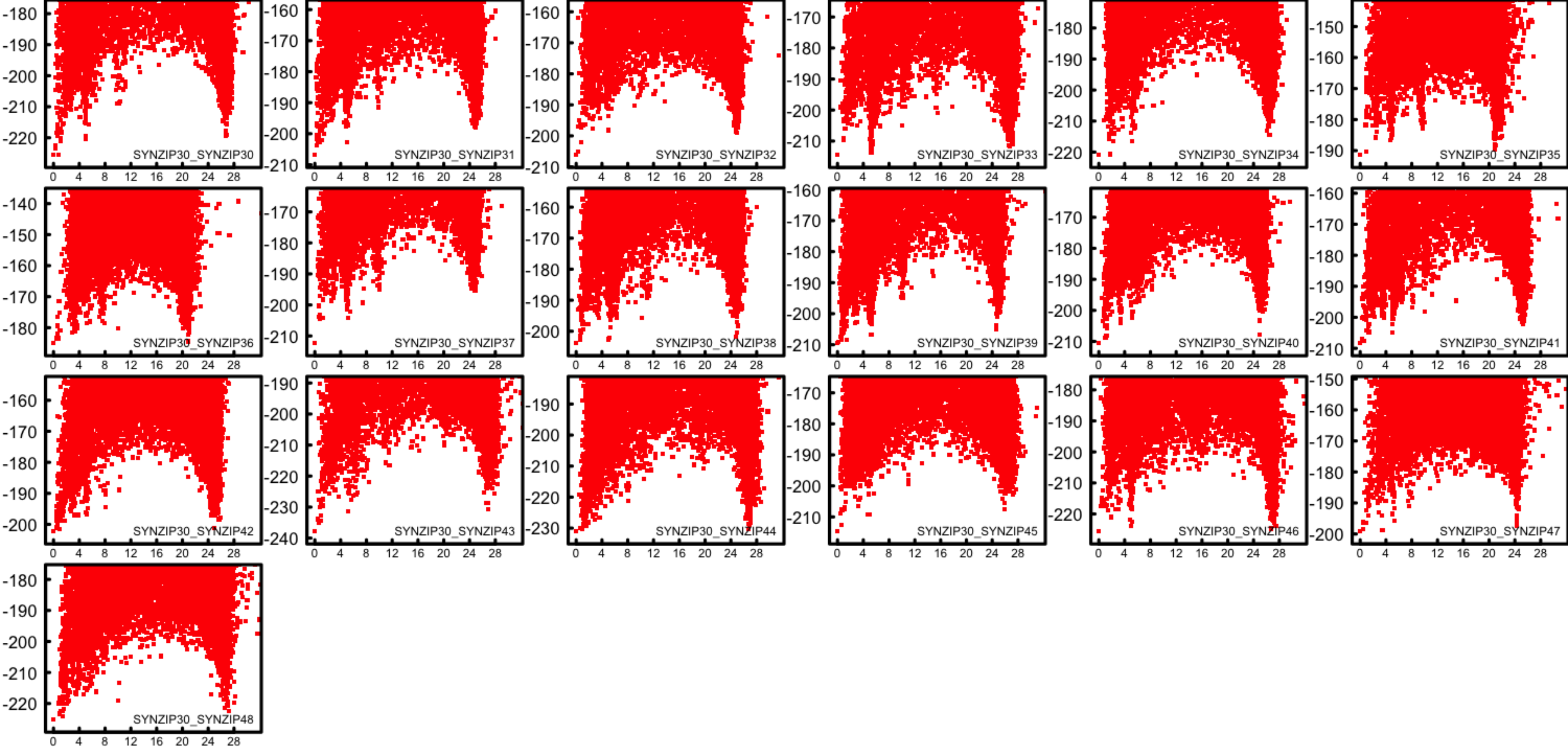

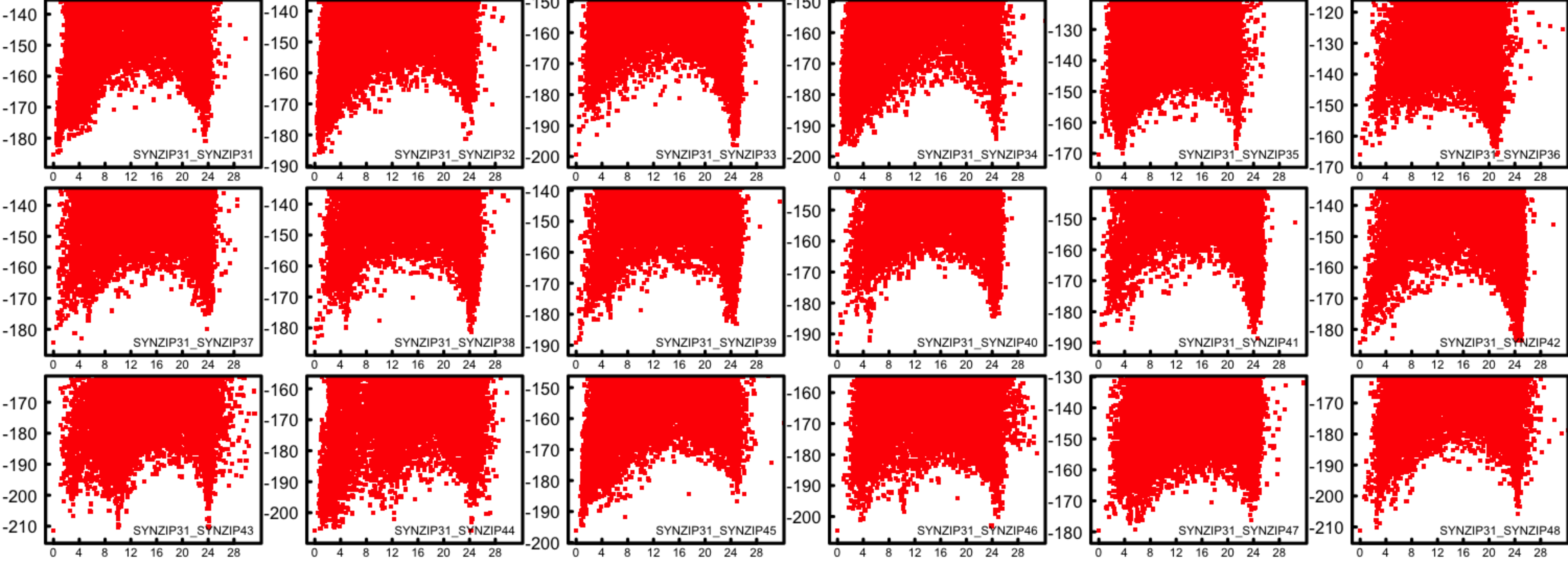

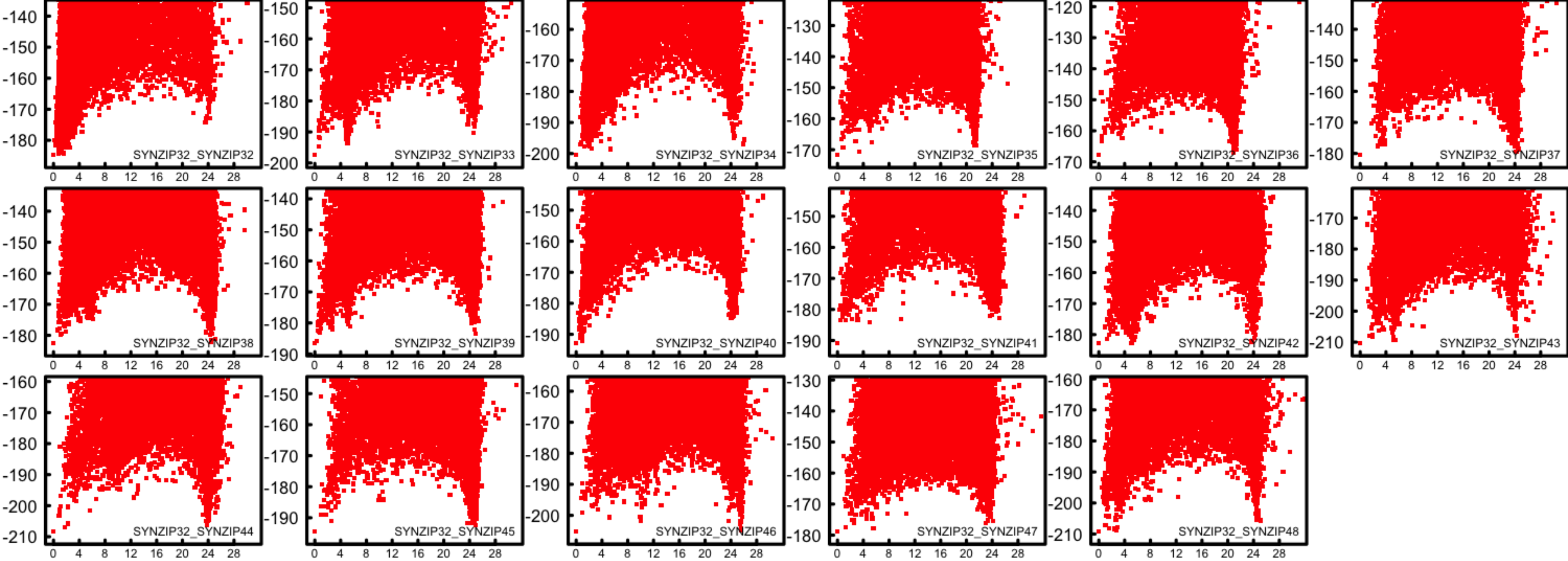

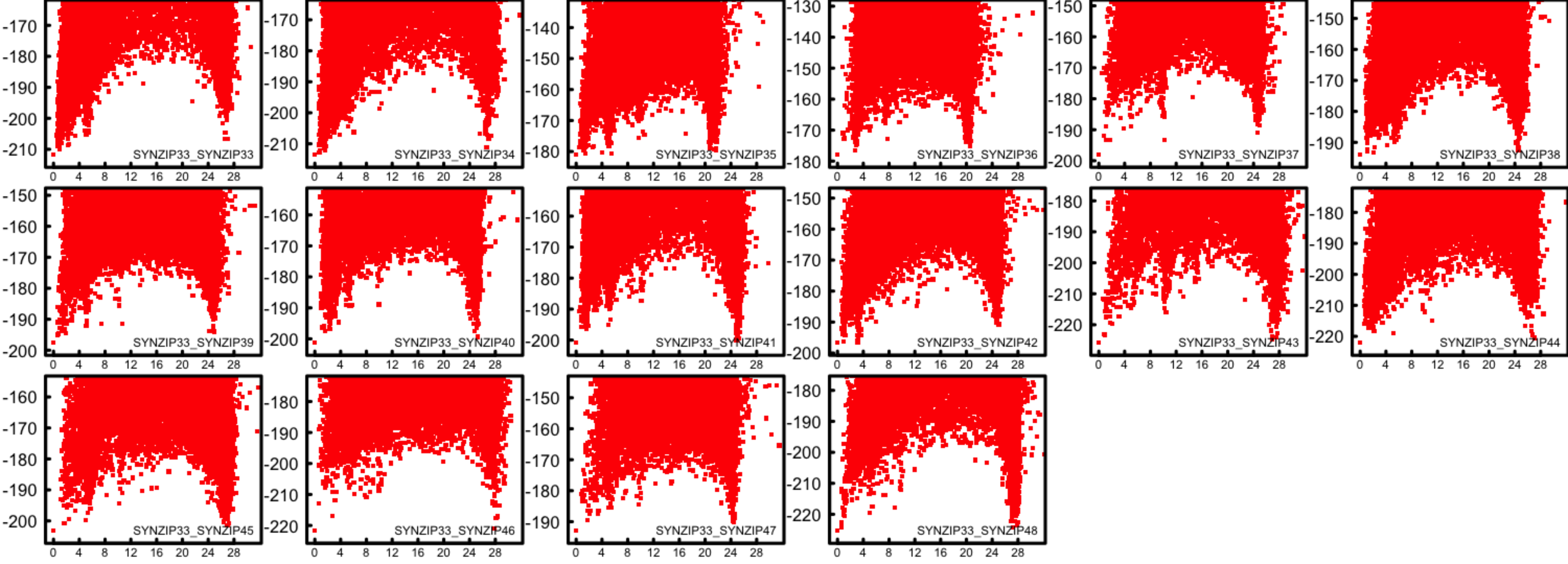

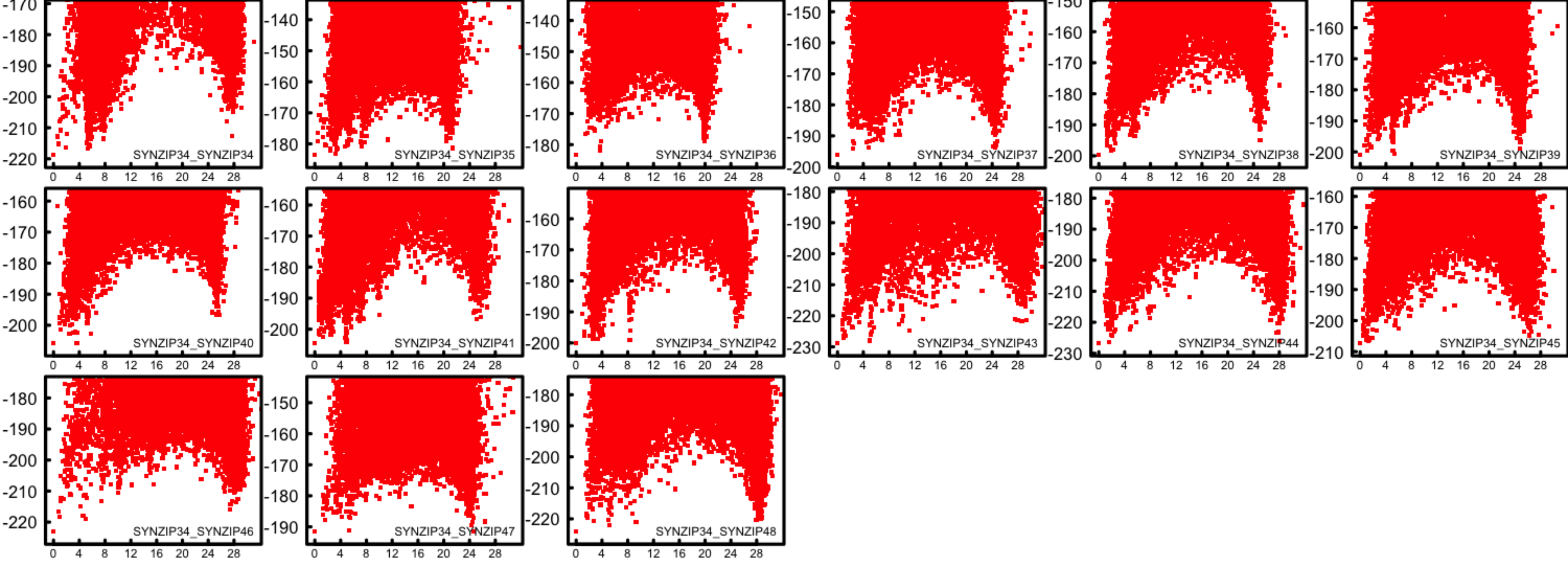

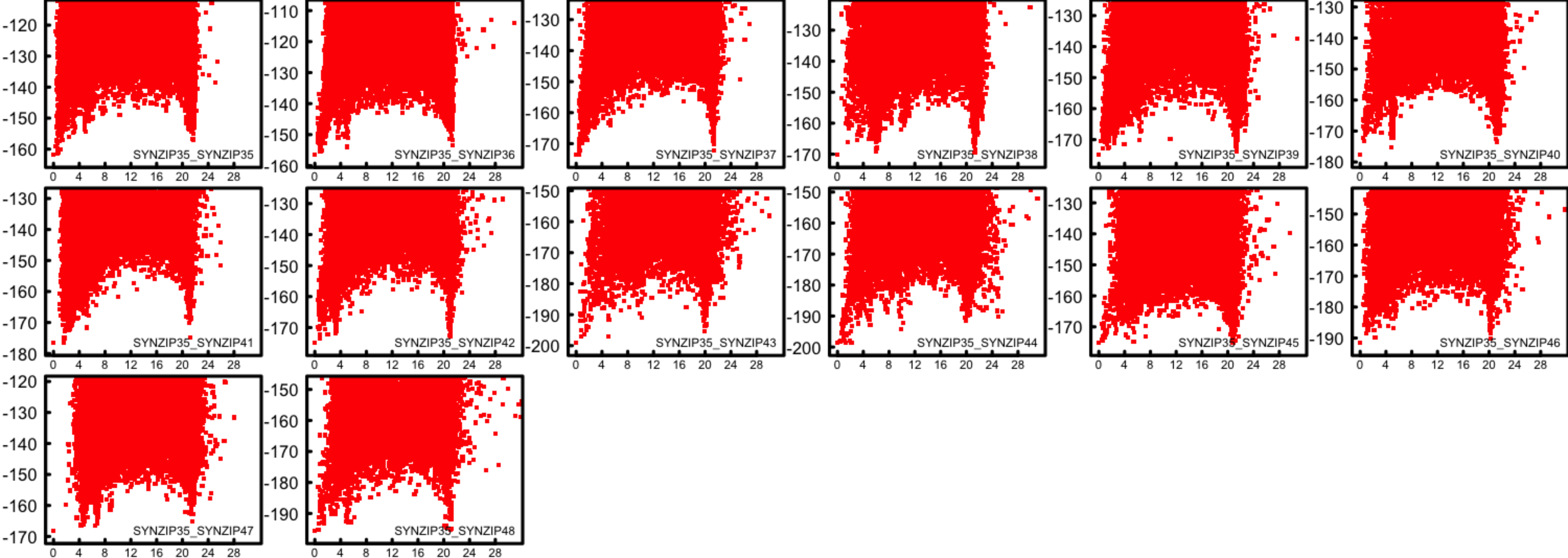

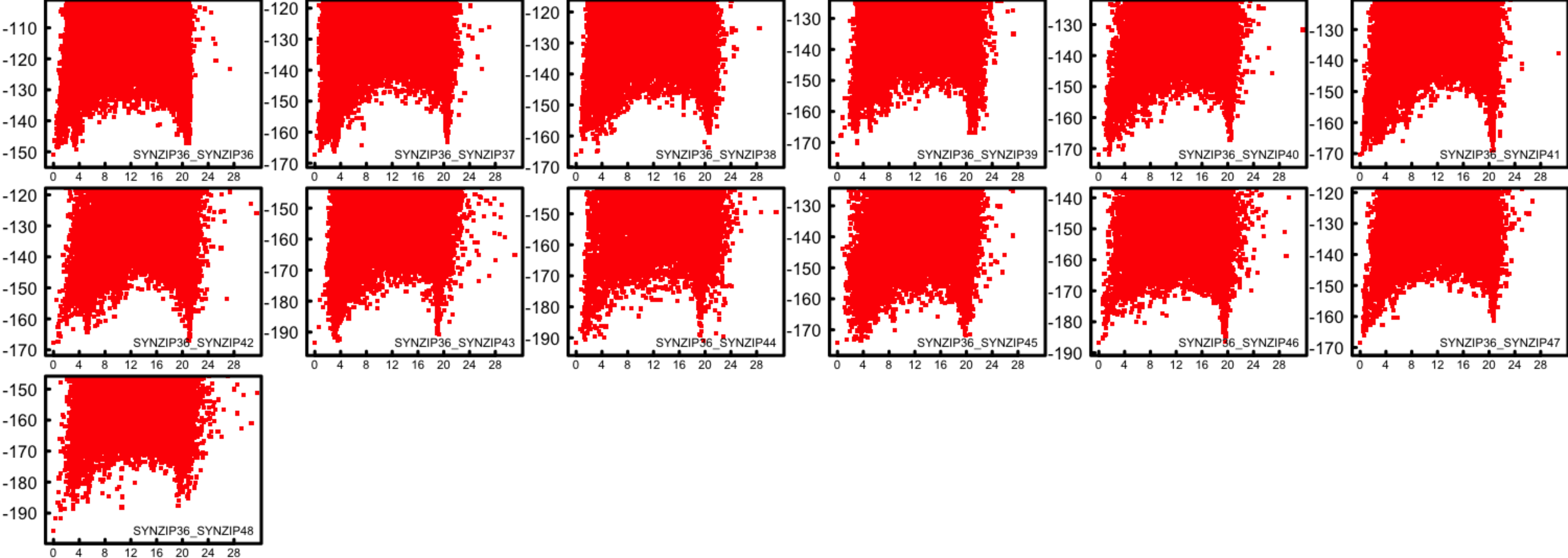

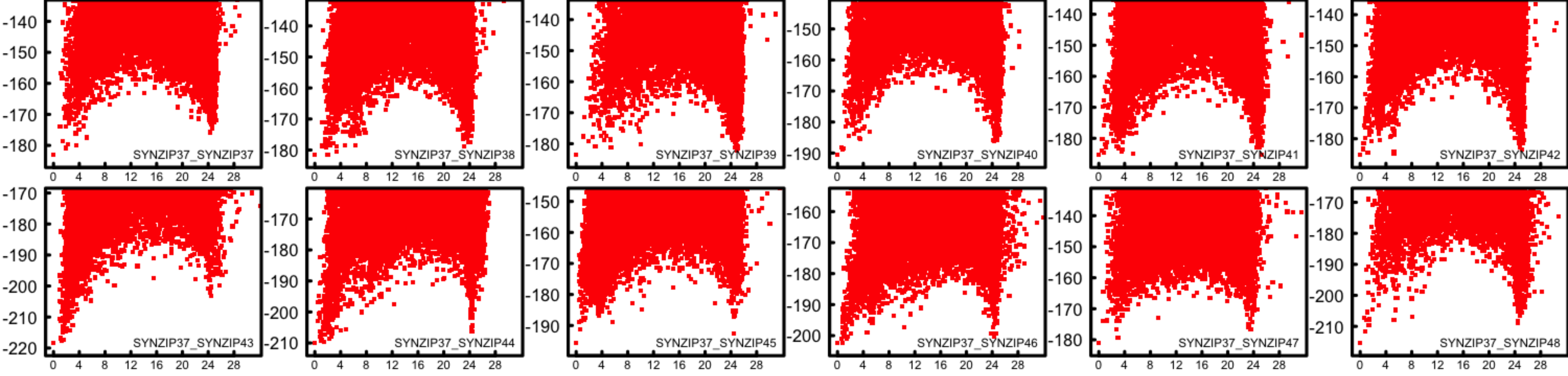

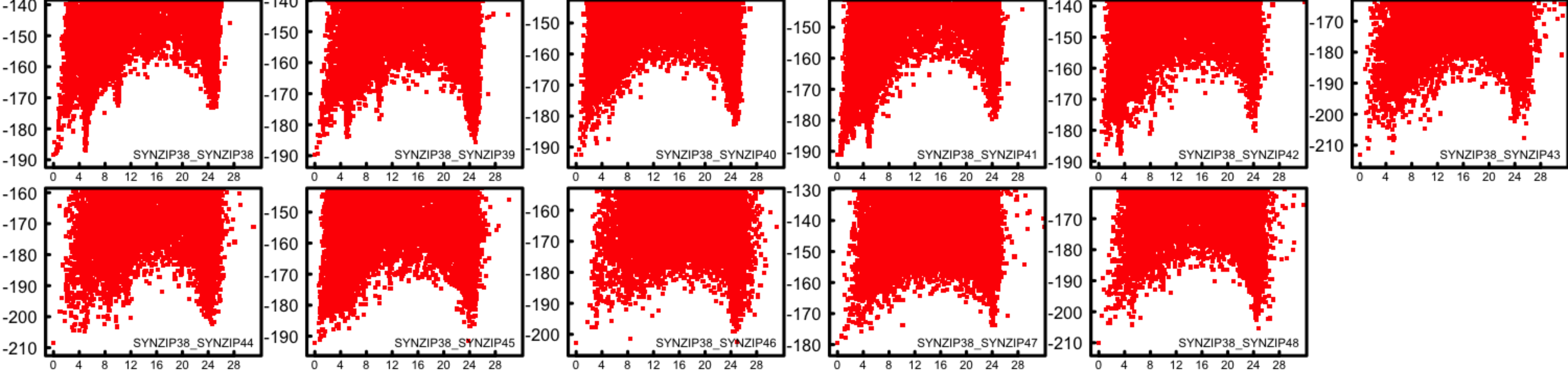

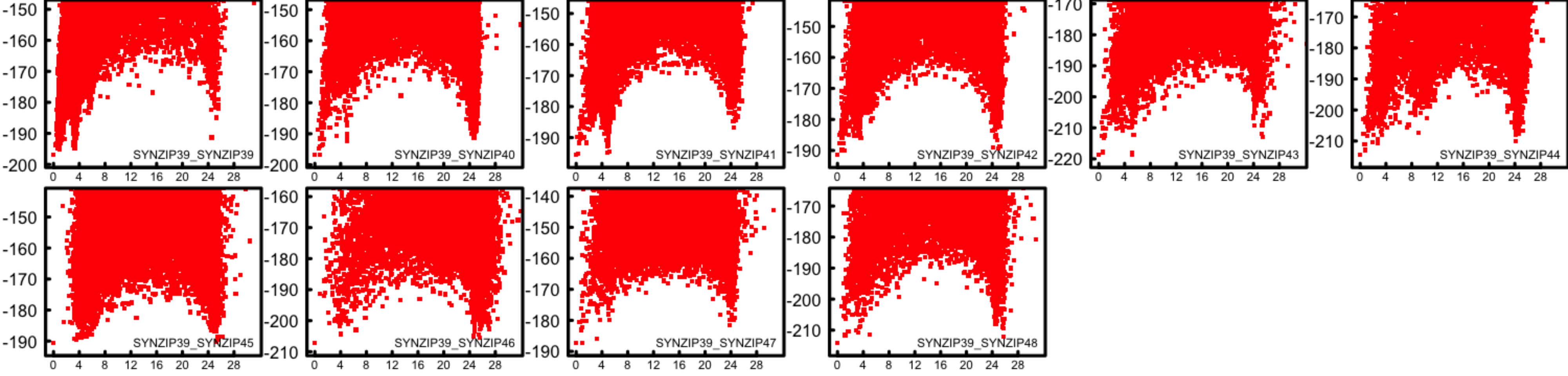

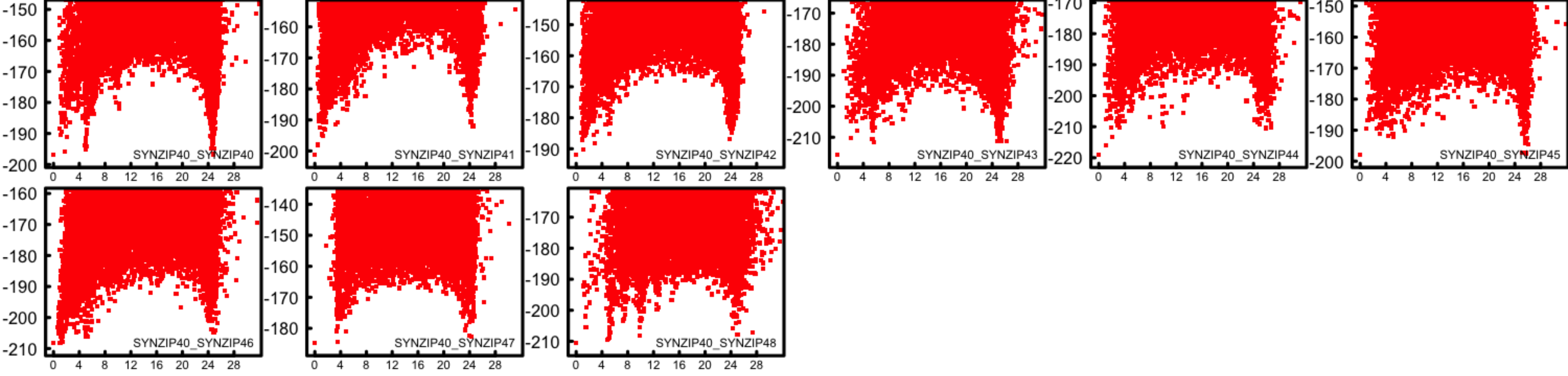

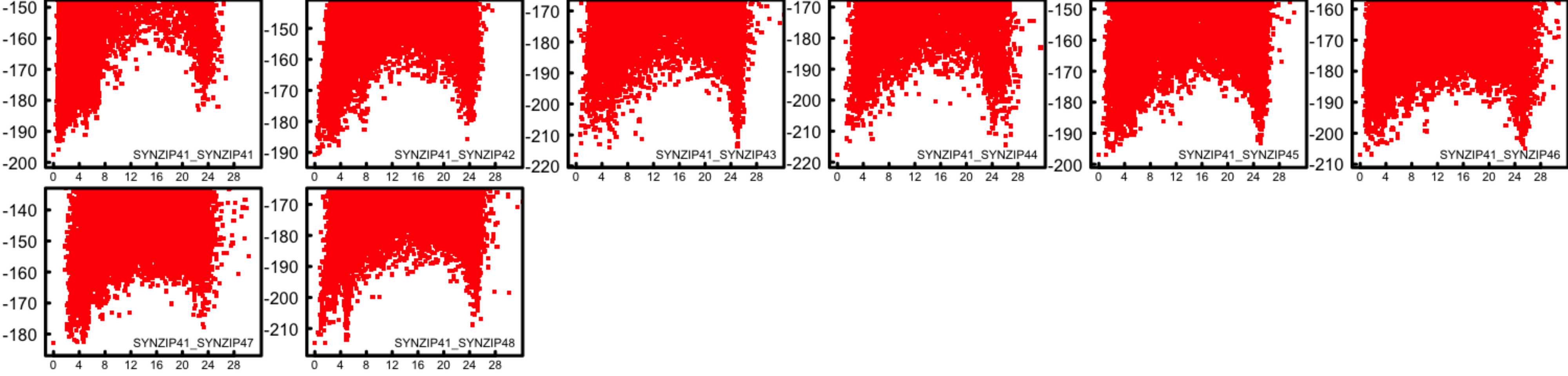

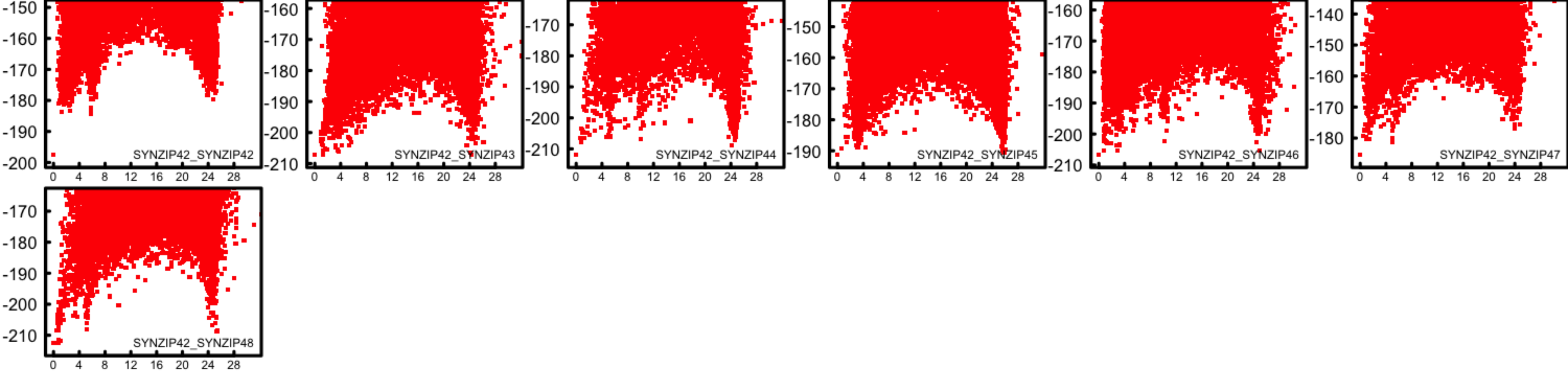

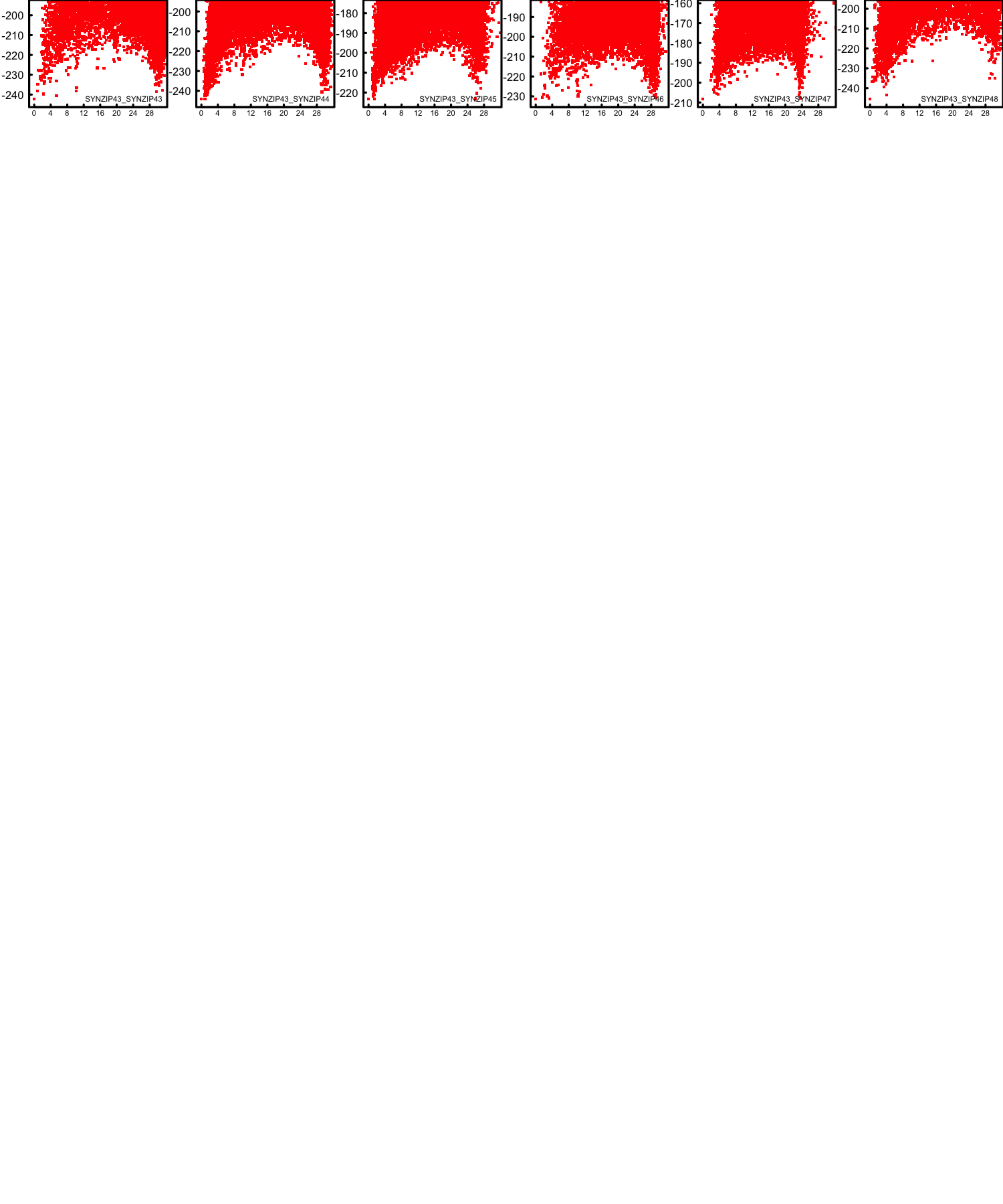

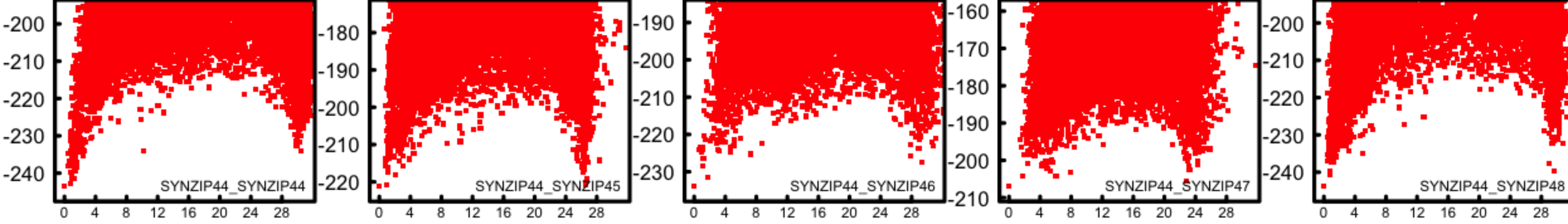

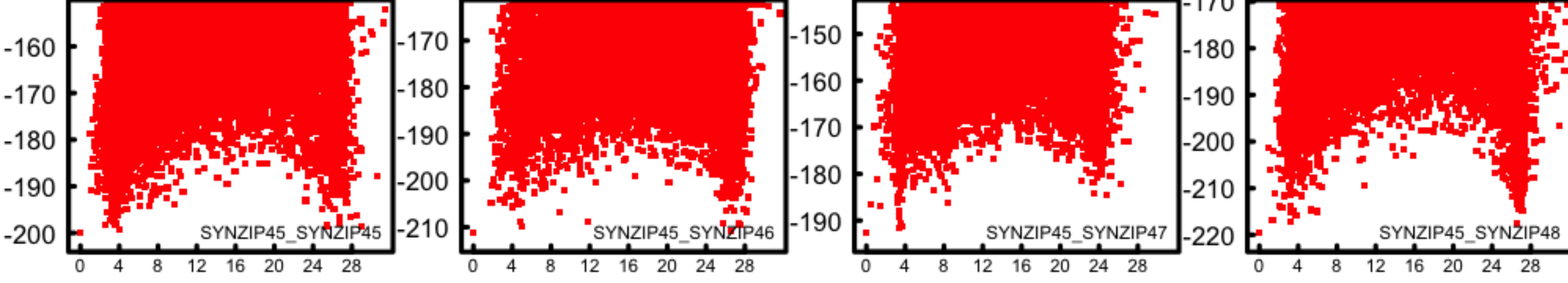

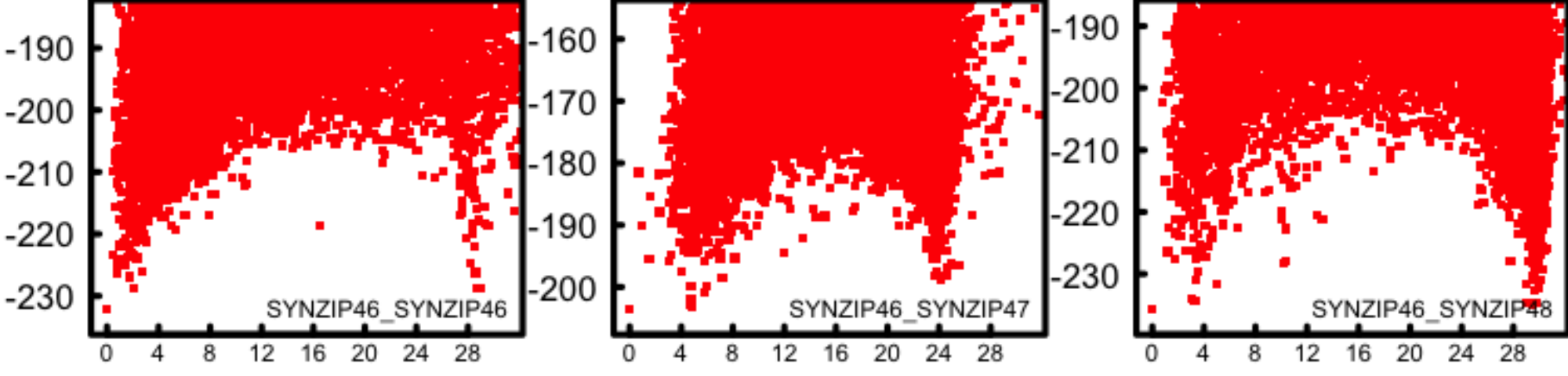

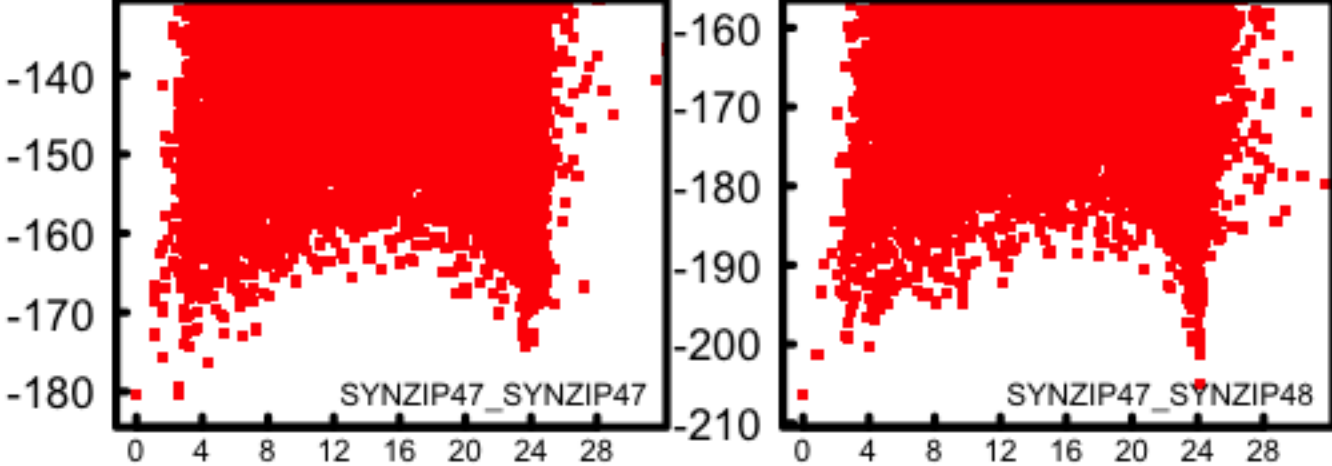

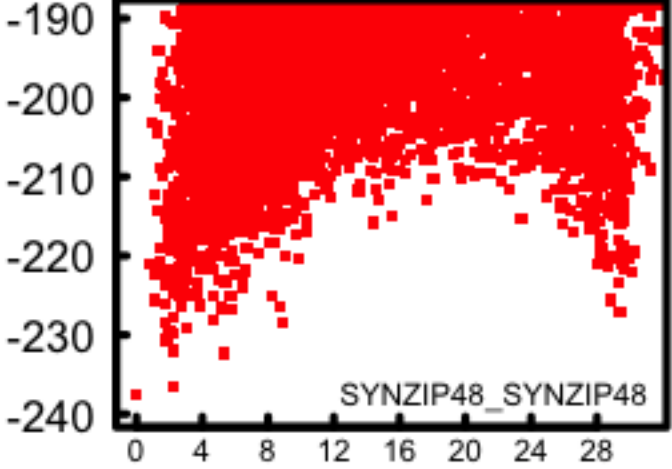

Supplement: Supplementary file 1 [file ijms-22-01368-s001.pdf]
